# Total Polysaccharides of Lily Bulb Ameliorate Menopause-Like Behavior in Ovariectomized Mice: Multiple Mechanisms Distinct from Estrogen Therapy

**DOI:** 10.1155/2019/6869350

**Published:** 2019-07-25

**Authors:** Xi-Dan Zhou, Yu Zheng, Rakesh Sharma, Zhang-Jin Zhang

**Affiliations:** ^1^School of Chinese Medicine, LKS Faculty of Medicine, The University of Hong Kong, Hong Kong, China; ^2^Proteomics & Metabolomics Core Facility, LKS Faculty of Medicine, The University of Hong Kong, Hong Kong, China

## Abstract

Our previous study has demonstrated the effects of aqueous extract of lily bulb in alleviating menopause-related psychiatric symptoms in ovariectomized (OVX) mice. This study sought to further investigate the psychotropic effects of total polysaccharides of lily bulb (TPLB) against anxiety, depression, and cognitive deterioration and the underlying mechanisms in OVX mice using behavioral, neurochemical, molecular, and proteomic approaches in comparison with estrogen therapy. While TPLB and estradiol showed similar effects in reducing OVX-induced anxiety, depression, and cognitive impairment, the psychotropic effects of TPLB were more closely associated with the predominant activation of estrogen receptors (ERs) and regulation of brain regional neurotransmitters and neurotrophins with minor effects on the uterus. Estradiol had similar potencies in binding affinity at ER*α* and ER*β*, which caused widespread genetic and epigenetic effects. In contrast, TPLB displayed a higher affinity at ER*β* than ER*α*, triggering the specific Ras/Akt/ERK/CREB signaling pathway without affecting any epigenetic activity. TPLB additionally modulated multiple proteins associated with mitochondrial oxidative stress, but estradiol did not. These results indicate that TPLB has comparable efficacy in reducing menopause-associated neuropsychological symptoms with a better safety profile compared to estrogen therapy. We suggest that TPLB could serve as a novel agent for menopause syndrome.

## 1. Introduction

Menopause, a natural physiological process for women, refers to a series of dysfunctions in the autonomic nervous system due to hormone fluctuation induced by ovarian failure, accompanied with neuropsychological symptoms, such as anxiety, depression, and cognitive deterioration [1, 2]. These psychological disorders have a negative impact on the quality of life of menopausal women in varying degrees especially taking the increased life expectancy into consideration [3]. Estrogen replacement therapy is currently the most effective treatment option for alleviating most menopausal syndromes [4], but a large body of evidence confirms that long-term estrogen therapy increases the risk of breast and ovarian cancer, stroke, and cardiovascular disease [5, 6]. Therefore, a search of complementary and alternative therapy particularly from herbal medicine is highly desired [7].

Most recently, we have demonstrated that aqueous extract of lily bulb had comparable effects with estrogen therapy in alleviating menopause-associated psychiatric disorders and the psychotropic effects were achieved via the predominant protection of neurotransmitters, neurotrophins, and ER*β* in the brain [8]. As a well-investigated constituent of lily bulb, polysaccharides have been found to have antioxidative [9, 10], immunomodulatory [11, 12], antitumor [13, 14], and hypoglycemic [15] activities. In the current study, we hypothesized that total polysaccharides of lily bulb (TPLB) may also exert the ameliorative effects on menopause-associated psychiatric symptoms. To test this hypothesis, we investigated the effects of TPLB behavioral, neurochemical, molecular, and proteomic approaches and explored the underlying mechanisms associated with neurotransmitters, neurotrophins, and estrogen receptors (ERs).

The activation of the nuclear receptors ER*α* and ER*β* modifies the transcription of hundreds of target genes, resulting in the alteration of protein synthesis and ending up with numerous physiological responses of each estrogen-targeted tissue [16]. The ER antagonist ICI182,780 was used to detect whether the effects of TPLB on brain regional neurotransmitters and neurotrophins were mediated via estrogen receptors. The shotgun proteomic analysis was additionally used to compare the effects of TPLB and estrogen therapy in modulating potential signaling pathways.

## 2. Materials and Methods

### 2.1. Animals

All experimental procedures were approved by the Committee on the Use of Live Animals in Teaching and Research of the University of Hong Kong (CULATR 3812-15). Female C57BL/6N mice weighing 18 to 22 g at 8 weeks of age were purchased from Charles River Laboratory (Wilmington, MA, USA). Mice were housed at a constant temperature (23 ± 2°C) and maintained on a 12 h/12 h light/dark cycle (lights on 7:00-19:00) with ad libitum access to food and water. All mice were acclimatized for 1 week before the experiment.

**Figure 1:**
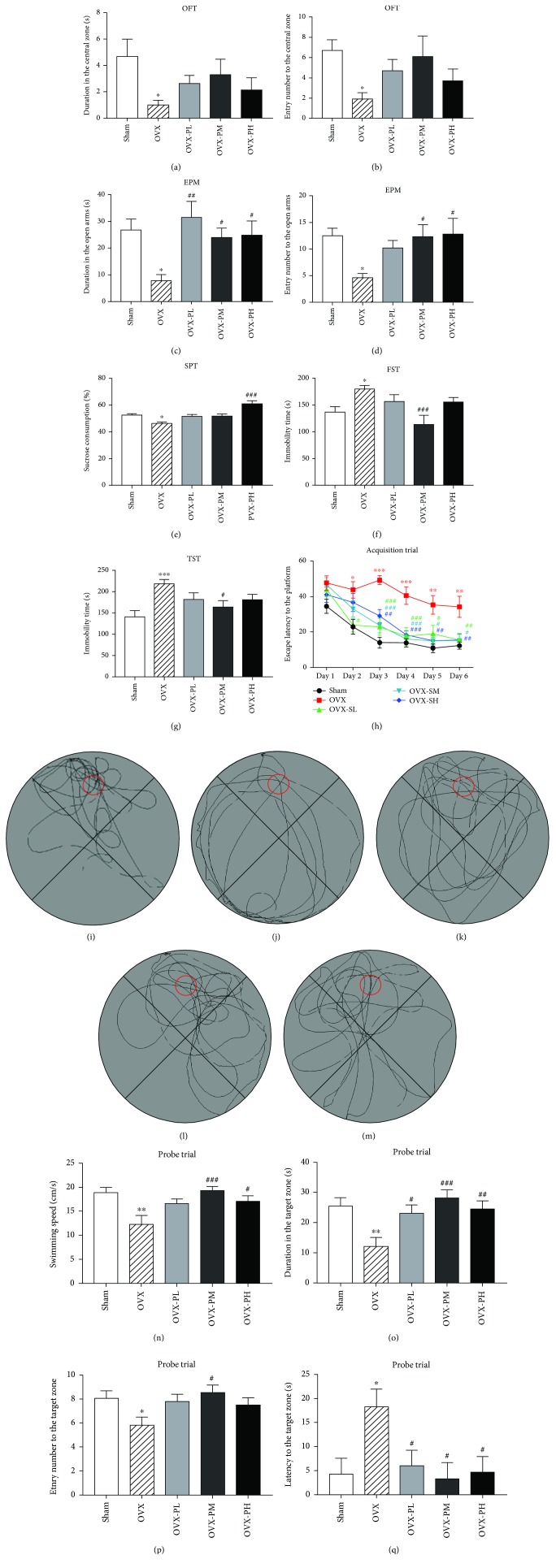
The effects of three doses of TPLB on OVX-induced anxiety- and depression-like behavior and cognitive performance. (a) Duration in the central zone in the open-field test (OFT); (b) number of entries into the central zone in the OFT; (c) duration in open arms in the elevated plus maze (EPM) test; (d) number of entries into the open arms in EPM; (e) percentage of sucrose consumption in the sucrose preference test (SPT); (f) immobility time of forced swimming test (FST); (g) immobility time of the tail suspension test (TST). Data are expressed as mean ± SEM (*n* = 10) and examined with one-way ANOVA, followed by post hoc Dunnett's test. (h) Escape latency in the acquisition trials; (i–m) representative individual swim paths in the probe trial of the sham group (i), OVX group (j), OVX-PL group (k), OVX-PM group (l), OVX- PH group (m); (n) swimming speed in the probe trial; (o) duration in the target zone in the probe trial; (p) entry number into the target zone in the probe trial; (q) latency to the target zone in the probe trial. Data are expressed as mean ± SEM (*n* = 10) and examined with two-way analysis of covariance (ANCOVA) and one-way ANCOVA, followed by post hoc Dunnett's test: ^∗^ *P* < 0.05, ^∗∗^ *P* < 0.01, and ^∗∗∗^ *P* < 0.001 vs. the Sham group; ^#^ *P* < 0.05, ^##^ *P* < 0.01, and ^###^ *P* < 0.001 vs. the OVX group. Different color symbols in (h) indicate the corresponding groups.

Experimental design is illustrated in S-Fig.  1. After one week of acclimatization, mice received ovariectomy (OVX) or sham surgery and were allowed to recover for 2 weeks. Chronic unpredictable mild stress (CUMS) procedure was performed on sham and OVX mice in the subsequent 2 weeks, followed by multiple behavioral tests. All agents were given immediately after OVX and throughout 36 days.

### 2.2. Ovariectomy (OVX)

Mice were anesthetized by intraperitoneal injection of a ketamine (10 mg/kg) and xylazine (10 mg/kg) mixture. Ovariectomies were performed via small bilateral dorsal flank incisions and subsequent removal of ovaries at 9 weeks of age. Sham-operated mice received similar incisions without ovary removal.

### 2.3. CUMS Procedure

Due to the fact that OVX was not sufficient to induce anxiety- and depression-like behavior as observed in our preliminary experiments, CUMS was thus added to mimic pressure experienced by menopausal women. CUMS was conducted at 2 weeks after OVX and throughout 2 weeks as we have done previously [17]. Multiple different types of mild stressors were carried out: tail clamping for 1 min, water deprivation for 15 h, food deprivation for 15 h, restraint in a plastic tube for 4 h, cage tilting at 45 degree for 15 h, empty cage without nesting for 15 h, illumination in a dark phase, and wet bedding (50 g sawdust/200 mL water) for 15 h. The mice received one of these stressors per day for two weeks, and the same stressor was not applied for 2 consecutive days to minimize the predictability of the occurrence of each stressor.

### 2.4. Preparation of Total Polysaccharides of Lily Bulb (TPLB)

The dry raw material derived from the roots of lily bulb (*Lilium lancifolium* Thunb.) was obtained from the Pharmacy of School of Chinese Medicine at the University of Hong Kong. Voucher specimens were identified by Dr. Yan-Bo Zhang, a senior Chinese herbalist, and deposited in the School of Chinese Medicine at the University of Hong Kong. To be consistent with our previous study, lily bulb was extracted with a 10-fold volume of distilled water for 2 h for three times. The extractive solution was pooled, centrifugated, and evaporated to about 300 mL concentrated extract, which was added with 4 volumes of 95% ethanol (*v*/*v*) for precipitation overnight. The precipitates were collected and dissolved in distilled water again, and the solution was deproteinized with the Sevag method [18] six times until there were no precipitations after added with Sevag solvent. The concentrated polysaccharide extract was added with 4 volumes of 95% ethanol (*v*/*v*) for precipitation again, and the obtained precipitate was washed with diethyl ether and lyophilized, which can be considered to contain the total polysaccharides. The yield of total polysaccharides is 6.12%. The content of total polysaccharides was measured using the phenol-sulfuric acid assay [19] with glucose (Sigma-Aldrich, St. Louis, MO) as standard, and the content of total polysaccharides was 87.3%.

### 2.5. Experimental Groups and Drug Administration

The animal experiments were performed by two steps. At the first step, the effects of TPLB at three doses on the OVX-induced depression, anxiety, and cognitive deterioration were investigated. 50 mice were randomly divided into five groups (*n* = 10 per group), including the sham-operated group (receiving vehicle only), OVX group (receiving vehicle only), OVX+PL group (receiving 50 mg/kg/day TPLB), OVX+PM group (receiving 100 mg/kg/day TPLB), and OVX+PH group (receiving 200 mg/kg/day TPLB). Based on our previous study, TPLB was also dissolved with vehicle (0.15% ethanol). All agents were given via oral gavage on a daily basis. At the second step, the ER antagonist ICI182,780 was used to evaluate whether the psychotropic effects of TPLB were mainly mediated by activating the ERs. 60 mice were randomly divided into six groups (*n* = 10 per group), including the sham-operated group (receiving vehicle only), OVX group (receiving vehicle only), OVX+E_2_ group (receiving 0.3 mg/kg/day Estradiol), OVX+E_2_+A group (receiving 0.3 mg/kg/day estradiol and 4 mg/kg/day ICI182,780), OVX+TP group (receiving 100 mg/kg/day TPLB), and OVX+TP+A group (receiving 100 mg/kg/day TPLB and 4 mg/kg/day ICI182,780). The determination of E_2_ dose was first based on the previous literature data [20–22] and then confirmed in our previous study [8]. ICI182,780 (Mayer Chemical Technology Co. Ltd, Shenzhen, China) was dissolved in DMSO in stock (400 mg/mL) and then diluted with distilled water to a concentration of 0.4 mg/mL at which the DMSO concentration of 0.1% yielded served as vehicle. The mice in the OVX+E_2_+A group and OVX+TP+A group were intraperitoneally administered with ICI182,780 at 9:00-10:00 am, while the remaining groups received 0.1% DMSO in the meantime. At 13:00-14:00 pm, the mice were treated with 0.15% ethanol, 0.3 mg/kg estradiol, and 100 mg/kg TPLB, respectively, via oral gavage. All agents were given on a daily basis.

### 2.6. Behavioral Tests

According to our previous protocol [17, 23], an open-field test (OFT) and an elevated plus maze (EPM) test were used to measure anxiety behavior. A sucrose preference test (SPT), a forced-swimming test (FST), and a tail suspension test (TST) were used to measure depressive behavior. A Morris water maze test was used to measure cognitive performance. The detailed procedures were described in Supplementary Materials.

### 2.7. Measurement of Serum Estradiol Level

Following the behavioral tests, 0.5 to 0.6 mL of blood was collected from each mouse via cardiac puncture. Sera were immediately separated by centrifuging at 3500 rpm for 15 min at 4°C and stored at -80°C until assay. The serum 17*β*-estradiol level was measured using a commercial enzyme-linked immunosorbent assay (ELISA) kit (Cloud-Clone Corp., Wuhan, China) according to the manufacturer's instructions.

### 2.8. Measurement of Brain Monoamine Neurotransmitters

After cardiac puncture, the mice were perfused with saline. Then, their brains were rapidly removed on ice, and three regions were separated, including the hypothalamus, hippocampus, and prefrontal cortex. Glutamate (Glu) and gamma-aminobutyric acid (GABA) were measured using a HPLC system with a diode-array detector (DAD) after derivatization procedure according to the previous report [24]. Briefly, the separated hypothalamus, hippocampus, and prefrontal cortex were homogenized in 100-200 *μ*L of acetonitrile. The homogenate was centrifuged at 13,000 rpm at 4°C for 20 min, and the supernatant was collected and evaporated under a gentle stream of nitrogen. The dried residue was reconstituted in 50-100 *μ*L of Na_2_CO_3_-NaHCO_3_ buffer (pH 9.5). The same volume of dansyl chloride (10 mmol/L) was added and vortex mixed for derivatization procedure, and the mixture was incubated in the dark at 65°C for 25 min and cooled at room temperature. Then, the solution was centrifuged at 13,000 rpm for 20 min, and 10 *μ*L of the supernatant was directly injected into a Thermo 3000 series UPLC equipped with an ACE Excel 2 C18 column (100 mm × 2.1 mm × 1.7 *μ*m) and a diode-array detector. The mobile phase consisted of acetonitrile (A) and 0.6% acetic acid in water/0.008% triethylamine (B). The gradient program was developed with70-55% B for 0–20 min. The flow rate was kept at 0.4 mL/min, and the detective wavelength was selected at 254 nm. The representational chromatograms are shown in S-Fig.  22.

### 2.9. Western Blot Analysis

Western blot analysis was used to examine the effects of TPLB on the estrogen receptors *α* and *β*, as well as three neurotrophic factors in the brain regions and the uterus. Furthermore, we also assessed the effects of TPLB on several biomarkers in the GABAergic, glutamatergic, serotonergic, and dopaminergic systems in the hypothalamus, hippocampus, and prefrontal cortex. Briefly, the separated tissues were homogenized in radioimmunoprecipitation assay buffer (RIPA buffer; Sigma-Aldrich, USA) containing 2% phenylmethanesulfonyl fluoride (PMSF; Sigma-Aldrich, USA). The supernatant was collected, and their protein concentration were measured with the Bradford method using Coomassie brilliant blue G-250 (Bio-Rad Laboratories Inc.). Proteins were separated by electrophoresis on 10-15% SDS-PAGE gels and subsequently transferred onto polyvinylidene difluoride membranes (PVDF; 0.22 *μ*M; Bio-Rad Laboratories Inc.). After being blocked with 5% BSA in TBST, the blots were probed with the antibodies listed in S-Table 1 at 4°C overnight. After rinsing with TBST, the membranes were incubated with suitable secondary antibodies (1 : 2000, Santa Cruz Biotechnology, USA) at 4°C for 4 h. Chemiluminescence was detected using an enhanced chemiluminescence detection kit (GE Healthcare, UK). The intensity of the bands was quantified by scanning densitometry using Image Lab 5.1 software (Bio-Rad Laboratories Inc.). The mean value of the intensity was obtained from at least three independent experiments.

### 2.10. Proteomic Analysis

The prefrontal cortex (PFC) from mice was suspended in the RIPA lysis buffer, and proteins were extracted from lysate using the Precellys homogenizer followed by centrifugation at 14,000 g for 30 min. Supernatant fraction was collected for protein quantitation using the BCA assay. Briefly, 100 *μ*g proteins were subjected to trypsin digestion following reduction and alkylation using the filter-aided sample preparation (FASP) method [25]. LysC-Tryptic peptides were cleaned using C18 stage tips and speedvac dried. Peptides are reconstituted in 0.1% formic acid for LC-MS/MS analysis to be carried out on an Orbitrap Fusion Lumos mass spectrometer interfaced with Dionex 3000RSLC nanoLC as described here [26].

Eluted peptides were analyzed with the Dionex Ultimate3000 nanoRSLC system coupled with Thermo Fisher Orbitrap Fusion Tribrid Lumos. Peptides were separated on a commercial C18 column (75 *μ*m i.d.×50 cm length) with 1.9 *μ*m particle size (Thermo Fisher). Separation was attained using a linear gradient of increasing buffer B (80% ACN and 0.1% formic acid) and declining buffer (0.1% formic acid) at 300 nL/min. Buffer B was increased to 30% B in 70 min and ramped to 40% B in 5 min followed by a quick ramp to 80% B, where it was held for 5 min before a quick ramp back to 5% B, where it was held, and the column was reequilibrated. Mass spectrometer was operated in positive polarity mode with capillary temperature of 300°C. Full MS survey scan resolution was set to 120,000 with an automatic gain control (AGC) target value of 2 × 106, maximum ion injection time (IT) of 50 ms, and a scan range of 350–1700 m/z. A data-dependent top 10 method was operated during which higher-energy collisional dissociation (HCD) was used. Spectra were obtained at 30,000 MS2 resolution with AGC target of 1 × 105 and maximum ion injection time (IT) of 80 ms, 1.6 m/z isolation width, and normalized collisional energy of 30. Preceding precursor ions targeted for HCD were dynamically excluded for 50 s. The high-resolution, high-mass-accuracy MS data obtained were processed using MaxQuant version 1.5.3.30, in which MS data analyzed in triplicates for each condition were searched using the Andromeda algorithm against UniProt Human protein database, appropriate parameter settings to obtain peptide and protein data using 0.1% FDR at peptide and protein level.

Proteins identified from OVX as compared to sham, E_2_, and TPLB-treated groups were quantified using the peptide LFQ intensities, and their obtained ratio was used for label-free quantitation to calculate the fold change (≥1.5-fold cutoff). Data visualization and statistical data analysis were performed by Perseus software version 1.5.4.1. Differential proteins were subjected to gene ontology (GO) enrichment analysis and KEGG (Kyoto Encyclopedia of Genes and Genomes) pathway analysis using the Database for Annotation, Visualization and Integrated Discovery (DAVID) Bioinformatics Resource 6.8 (https://david.ncifcrf.gov/) [27]. Secondary GO annotation mainly classifies the three major categories of protein functional activities: biological process (BP), cell component (CC), and molecular function (MF), and each protein was endowed with more than one functional annotation. PPI (Protein-Protein Interaction) Analysis was analyzed in STRING.

### 2.11. ER Competitive Ligand-Binding Assay

To confirm whether TPLB could bind to ERs, the PolarScreen™ ER Alpha and Beta Competitor Assay kits were used (Life Cat No.: A15883 and A15890). Briefly, gradient dilutions of TPLB (0.27, 0.8, 2.5, 7.4, 22.2, 66.7, 200, and 600 *μ*g/mL) were competed with fluorescent estrogen ligand for binding to ER *α* and *β* on a 384-well plate. 20 *μ*M estradiol was served as control ligand. Five hours after incubation at room temperature, the fluorescence polarization value was detected on SpectraMax iD5.

### 2.12. Statistical Analysis

**Figure 2:**
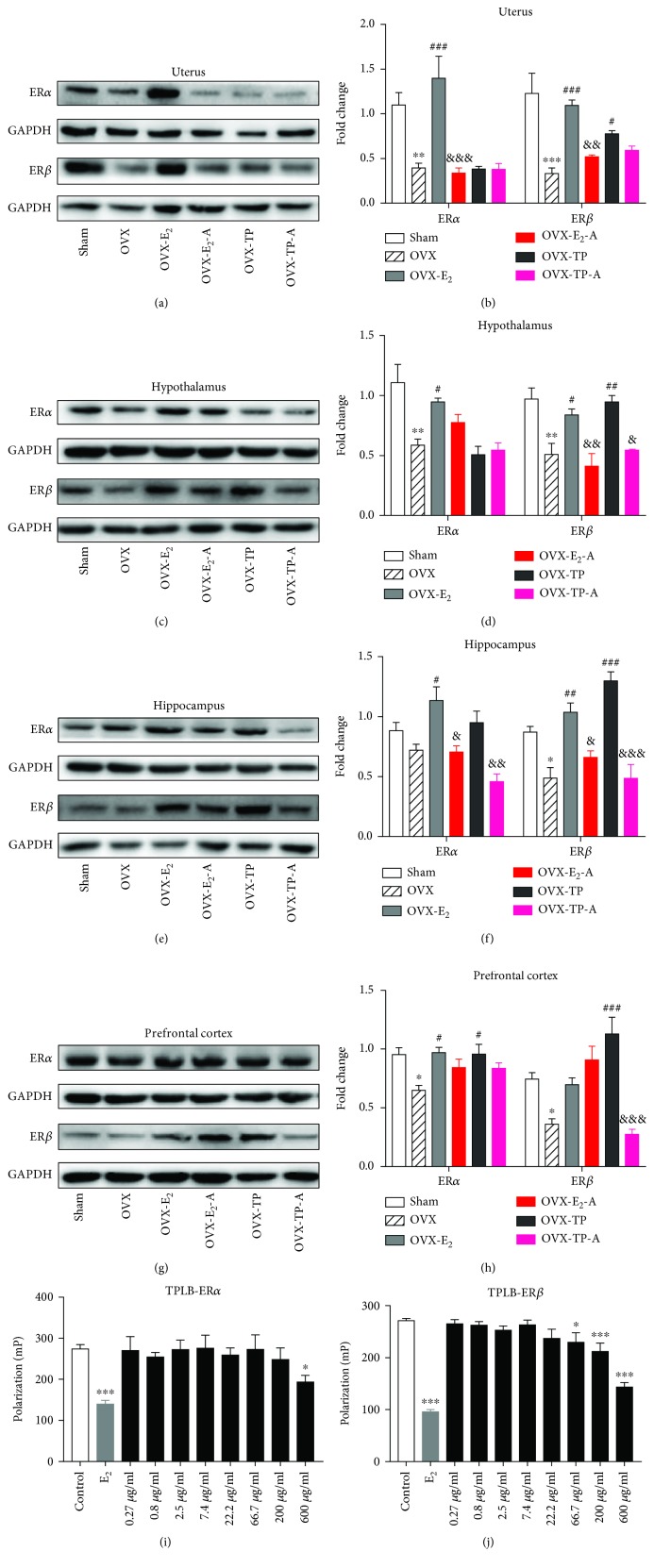
The effects of estradiol and TPLB on the expression levels of estrogen receptors, ER*α* and ER*β*, in the uterus and brain regions after coadministration with ICI182,780. Representative images indicating the expression levels of ER*α* and ER*β* and their quantification analysis in the uterus (a, b), hypothalamus (c, d), hippocampus (e, f), and prefrontal cortex (g, h). Data are expressed as mean ± SEM (*n* = 3) and examined with one-way ANOVA, followed by post hoc Sidak's multiple comparison test: ^∗^ *P* < 0.05, ^∗∗^ *P* < 0.01, and ^∗∗∗^ *P* < 0.001 vs. the sham group; ^#^ *P* < 0.05, ^##^ *P* < 0.01, and ^###^ *P* < 0.001 vs. the OVX group; ^&^ *P* < 0.05, ^&&^ *P* < 0.01, and ^&&&^ *P* < 0.001 vs. the E_2_ group or TP group. (i, j) The effects of TPLB on the ability of ER*α* binding (a) and ER*β* binding (b) in vitro. Data are expressed as mean ± SEM (*n* = 6‐10) and examined with one-way ANOVA, followed by post hoc Dunnett's test: ^∗^ *P* < 0.05, ^∗∗^ *P* < 0.01, and ^∗∗∗^ *P* < 0.001 vs. the control group.

Data were expressed as mean ± standard error of the mean (SEM). Due to a marked difference found in swimming speed among groups, two-way analysis of covariance (ANCOVA) and one-way ANCOVA were, respectively, used to detect the effects on training and probe trial variables of the water maze test with swimming speed as covariate. One-way analysis of variance (ANOVA) was used to examine other variables. Between-group differences were further analyzed using Dunnett's test or Sidak's test. Dunnett's test was used to compare each column against the default control column, while Sidak's test was used for analysis of differences among preselected groups. For Figures 1, 2(i) and 2(j), Dunnett's test was selected because all groups were compared against the OVX group (Figure 1) or E_2_ (Figures 2(i) and 2(j)). Sidak's test was used for those quantitative figures with ER antagonist ICI182,780 because comparisons were performed among multiple groups rather than the default control group. All statistical analysis was conducted with GraphPad Prism 7.0 software (La Jolla, CA). Statistical significance was defined as less than 0.05 of *P* value.

## 3. Results

### 3.1. Effects of Three Doses of TPLB on Anxiety-Like Behavior

As shown in Figures 1(a)–1(d), OVX caused a widespread decrease in the duration in and number of entries into the central zone (OFT) and the open arms (EPM). In the EPM test, the OVX mice treated with 100 mg/kg and 200 mg/kg TPLB showed a significant increase in the time spent in the open arms and number of entries into the open arms as compared to the vehicle-treated OVX group. The OVX mice receiving 50 mg/kg TPLB also spent more time in the open arms than those that received vehicle. In the OFT, the OVX mice treated with three doses of TPLB showed no significant effects on the duration in and entry number into the central zone. However, the OVX mice treated with PM showed a marked trend in increasing the entry number to the central zone in OFT (*P* = 0.0788), although it did not reach the significance level (*P* < 0.05) due to the large bar.

### 3.2. Effects of Three Doses of TPLB on Depression-Like Behavior

The OVX mice showed a marked decrease in sucrose consumption in the SPT and a significant increase in the immobility time spent in the FST and TST compared with the sham-operated mice in Figures 1(e)–1(g). The OVX mice treated with TPLB at the dose of 100 mg/kg significantly reduced the immobility time spent in the FST and TST compared with OVX mice treated with vehicle. The OVX mice receiving 200 mg/kg TPLB consumed more sucrose than those vehicle-treated OVX mice.

### 3.3. Effects of Three Doses of TPLB on Cognitive Performance

In the training trials, vehicle-treated OVX mice took much longer latency to the platform than mice with sham surgery from Day 2 to Day 6. The latency of OVX mice treated with three doses of TPLB was significantly shorter than that of those treated with vehicle at Day 3 through Day 6.

In the probe trial, OVX significantly reduced the time spent in and the number of entries into the target zone and increased the latency to the target zone compared to sham surgery (Figures 1(n)–1(q)). The OVX mice treated with 100 mg/kg TPLB had more duration in, more frequency crossed, and shorter latency to the target zone than those treated with vehicle. The OVX mice treated with 50 mg/kg and 200 mg/kg TPLB also showed a remarkable increase in time spent in the target zone and a significant decrease in the latency to the target zone. The representative navigation paths of the five groups are shown in Figures 1(i)–1(m).

**Figure 3:**
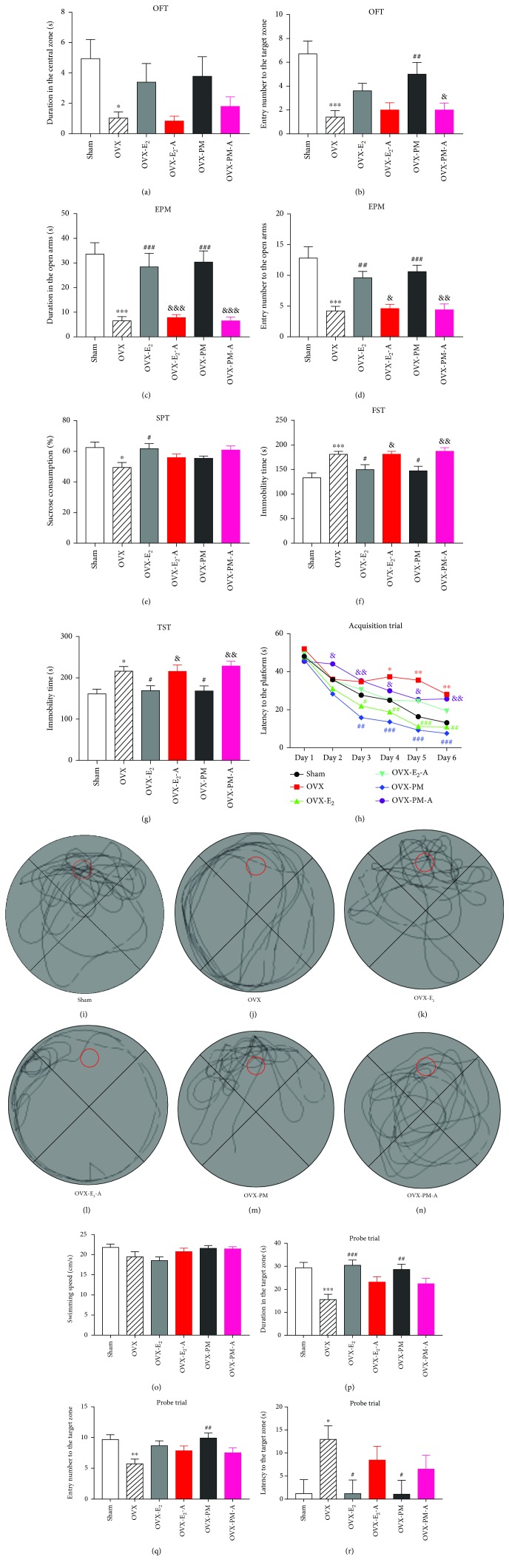
The effects of estradiol and TPLB on OVX-induced anxiety- and depression-like behavior and cognitive performance after coadministration with ICI182,780. (a) Duration in the central zone in the open-field test (OFT); (b) number of entries into the central zone in the OFT; (c) duration in open arms in the elevated plus maze (EPM) test; (d) number of entries into the open arms in EPM; (e) percentage of sucrose consumption in the sucrose preference test (SPT); (f) immobility time of forced swimming test (FST); (g) immobility time of the tail suspension test (TST). Data are expressed as mean ± SEM (*n* = 10) and examined with one-way ANOVA, followed by post hoc Sidak's multiple comparison test. (h) Escape latency in the acquisition trials; (i–n) representative individual swim paths in the probe trial of the sham group (i), OVX group (j), OVX-E_2_ group (k), OVX-E_2_-A group (l), OVX-TP group (m); OVX-TP-A group (n); (o) swimming speed in the probe trial; (p) duration in the target zone in the probe trial; (q) entry number into the target zone in the probe trial; (r) latency to the target zone in the probe trial. Data are expressed as mean ± SEM (*n* = 10) and examined with two-way analysis of covariance (ANCOVA) and one-way ANCOVA, followed by post hoc Sidak's multiple comparisons test: ^∗^ *P* < 0.05, ^∗∗^ *P* < 0.01, and ^∗∗∗^ *P* < 0.001 vs. the sham group; ^#^ *P* < 0.05, ^##^ *P* < 0.01, and ^###^ *P* < 0.001 vs. the OVX group; ^&^ *P* < 0.05, ^&&^ *P* < 0.01, and ^&&&^ *P* < 0.001 vs. the E_2_ group or TP group. Different color symbols in (h) indicate the corresponding groups.

**Figure 4:**
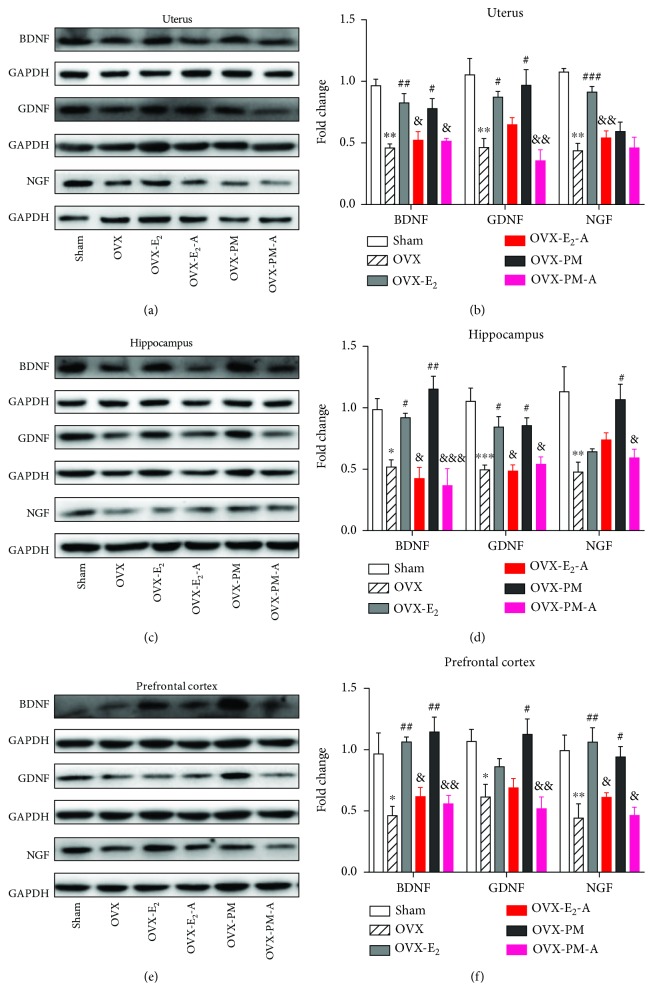
The effects of estradiol and TPLB on the expression levels of neurotrophins, BDNF, GDNF, and NGF, in the uterus, hippocampus, and prefrontal cortex after coadministration with ICI182,780. Representative images indicating the expression levels of three neurotrophins and their quantification analysis in the uterus (a, b), hippocampus (c, d), and prefrontal cortex (e, f). Data are expressed as mean ± SEM (*n* = 3) and examined with one-way ANOVA, followed by post hoc Sidak's multiple comparison test: ^∗^ *P* < 0.05, ^∗∗^ *P* < 0.01, and ^∗∗∗^ *P* < 0.001 vs. the sham group; ^#^ *P* < 0.05, ^##^ *P* < 0.01, and ^###^ *P* < 0.001 vs. the OVX group; ^&^ *P* < 0.05, ^&&^ *P* < 0.01, and ^&&&^ *P* < 0.001 vs. the E_2_ group or TP group.

**Figure 5:**
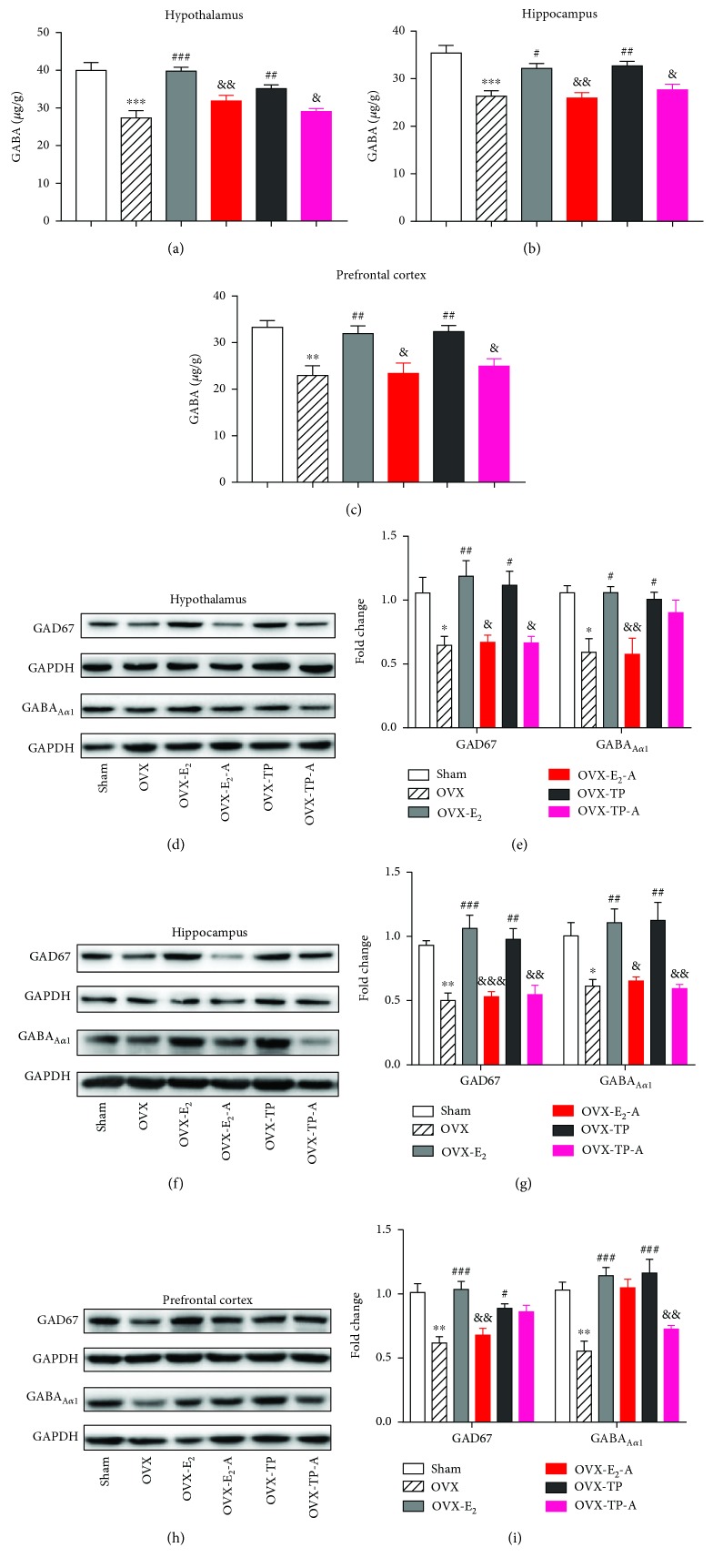
The effects of estradiol and TPLB on the GABAergic system in the brain regions. GABA levels (*n* = 5) in the hypothalamus (a), hippocampus (b), and prefrontal cortex (c); representative images indicating the expression levels (*n* = 3) of GABA_A*α*1_ and GAD67 and their quantification analysis in the hypothalamus (d, e), hippocampus (f, g), and prefrontal cortex (h, i). Data are expressed as mean ± SEM and examined with one-way ANOVA, followed by post hoc Sidak's multiple comparison test: ^∗^ *P* < 0.05, ^∗∗^ *P* < 0.01, and ^∗∗∗^ *P* < 0.001 vs. the sham group; ^#^ *P* < 0.05, ^##^ *P* < 0.01, and ^###^ *P* < 0.001 vs. the OVX group; ^&^ *P* < 0.05, ^&&^ *P* < 0.01, and ^&&&^ *P* < 0.001 vs. the E_2_ group or TP group.

**Figure 6:**
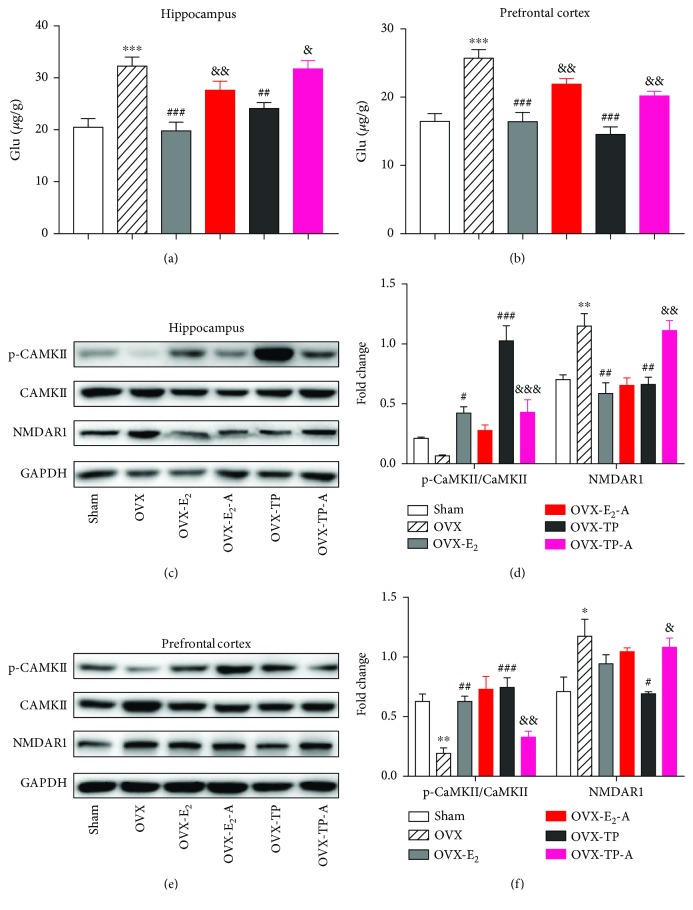
The effects of estradiol and TPLB on the glutamatergic system in the brain regions after coadministration with ICI182,780. Glutamate levels (*n* = 5) in the hippocampus (a) and prefrontal cortex (b); representative images indicating the expression levels (*n* = 3) of NMDAR1 and CaMKII and their quantification analysis in the hippocampus (c, d) and prefrontal cortex (e, f). Data are expressed as mean ± SEM and examined with one-way ANOVA, followed by post hoc Sidak's multiple comparison test: ^∗^ *P* < 0.05, ^∗∗^ *P* < 0.01, and ^∗∗∗^ *P* < 0.001 vs. the sham group; ^#^ *P* < 0.05, ^##^ *P* < 0.01, and ^###^ *P* < 0.001 vs. the OVX group; ^&^ *P* < 0.05, ^&&^ *P* < 0.01, and ^&&&^ *P* < 0.001 vs. the E_2_ group or TP group.

**Figure 7:**
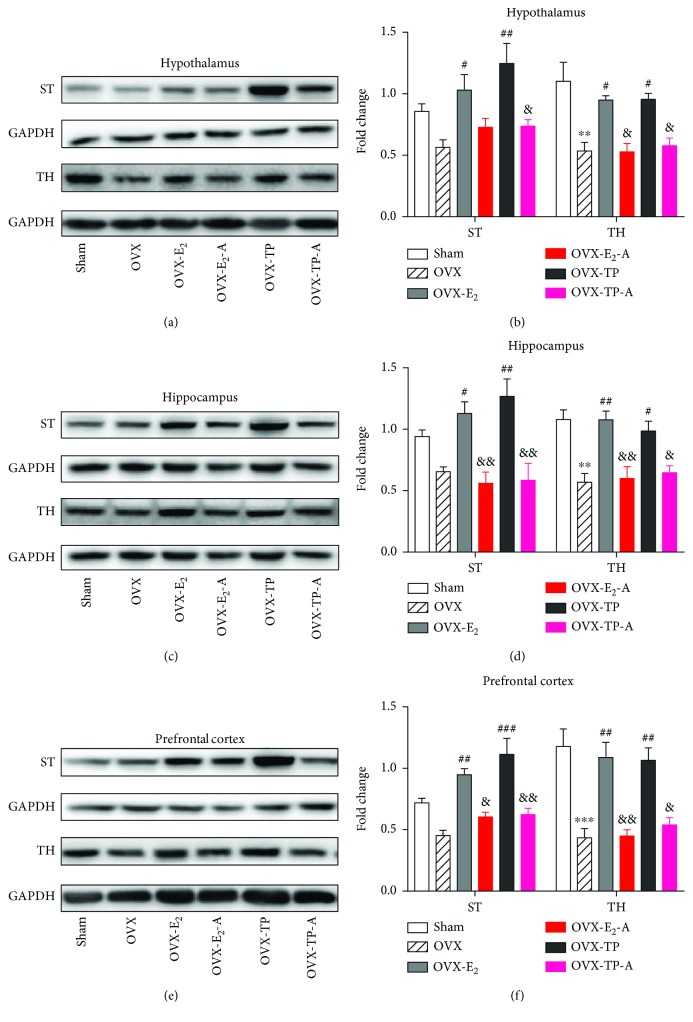
The effects of estradiol and TPLB on the serotonergic and catecholaminergic systems in the brain regions after coadministration with ICI182,780. Representative images indicating the expression levels of ST and TH and their quantification analysis in the hypothalamus (a, b), hippocampus (c, d), and prefrontal cortex (e, f). Data are expressed as mean ± SEM (*n* = 3) and examined with one-way ANOVA, followed by post hoc Sidak's multiple comparison test: ^∗^ *P* < 0.05, ^∗∗^ *P* < 0.01, and ^∗∗∗^ *P* < 0.001 vs. the sham group; ^#^ *P* < 0.05, ^##^ *P* < 0.01, and ^###^ *P* < 0.001 vs. the OVX group; ^&^ *P* < 0.05, ^&&^ *P* < 0.01, and ^&&&^ *P* < 0.001 vs. the E_2_ group or TP group.

**Figure 8:**
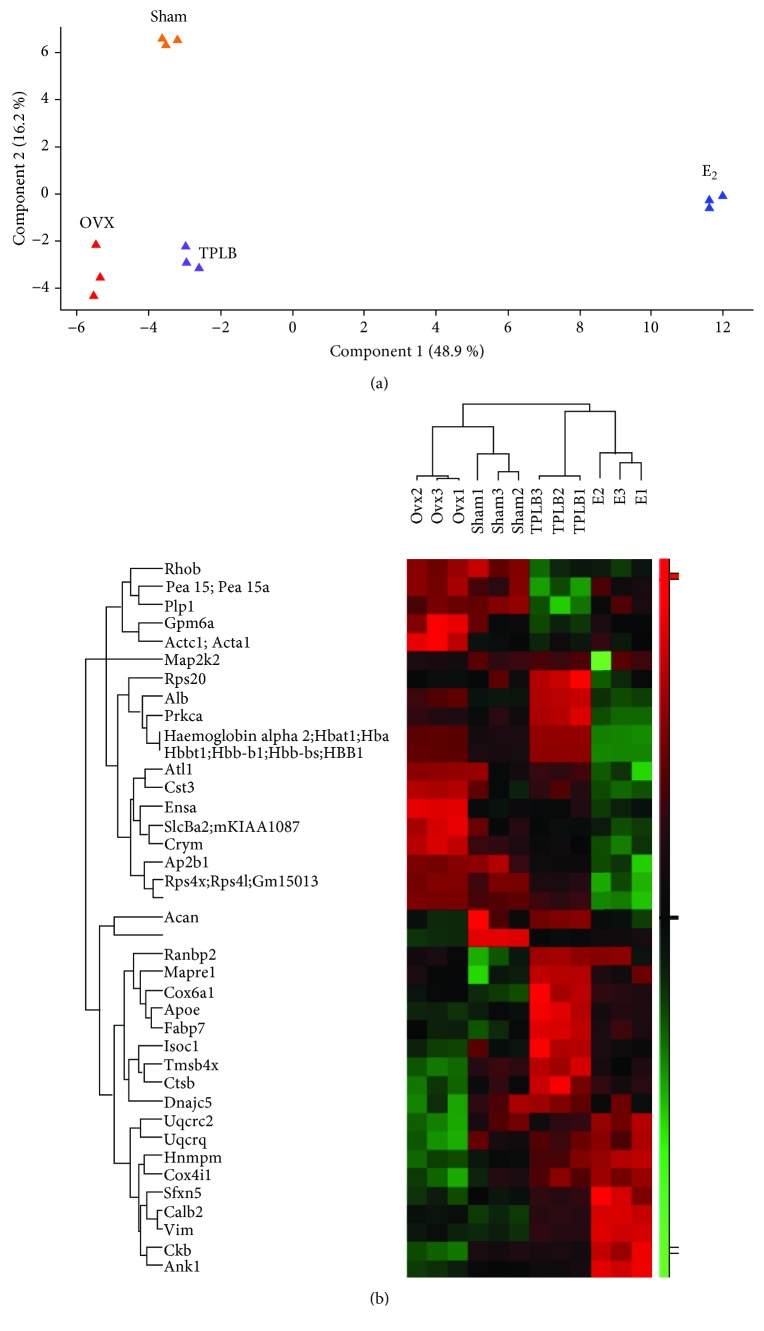
Clustering analysis: principal component analysis (a) and hierarchical clustering results (b) among sham, OVX, E_2_, and TPLB groups. Hierarchical clustering results were expressed as a tree heat map, with red representing upregulation and green indicating downregulation. *X*- and *Y*-coordinates represented sample and differentially expressed proteins, respectively.

In addition to the behavior tests, the effects of TPLB on the GABAergic, glutamatergic, serotoninergic, and catecholaminergic systems, neurotrophic systems, and estrogen receptors were described in Supplementary Materials (S-Fig.  3-7). However, TPLB had no effects on the OVX-induced weight gains, uterine shrinkage, and the drop of serum estrogen level (S-Fig.  1). Based on the results above, we selected the medium dose of 100 mg/kg TPLB for the following study. In the second study, the intervention of ER antagonist ICI182,780 completely blocked the effects of estradiol on OVX-induced weight gains, uterine shrinkage, and dramatic drop of serum estrogen level (S-Fig.  8).

### 3.4. Effects of Estradiol and TPLB on Anxiety-Like Behavior after Coadministration with ICI182,780

As indicated in Figures 3(a)–3(d), the OVX mice treated with estradiol and TPLB remarkably reversed the OVX-induced decreases in the time spent in the open arms and number of entries into the open arms as compared to the vehicle-treated OVX group. Different from Figure 1(b), treatment with 100 mg/kg TPLB significantly increased the entry number to central zone in this OFT, which was possibly attributed to the intergroup relatively consistent behavior and the resultant low bar. After coadministration with ICI182,780, the OVX mice showed remarkable decreases in the duration in and entry number into the open arms compared to those only treated with estradiol and TPLB in the EPM test. Additionally, ICI182,780 induced the significant decrease in the number of entries into the central zone, which was increased by TPLB treatment.

### 3.5. Effects of Estradiol and TPLB on Depression-Like Behavior after Coadministration with ICI182,780

The OVX mice treated with estradiol and TPLB significantly reduced the immobility time spent in the FST and the TST compared with OVX mice treated with vehicle (Figures 3(e)–3(g)). After coadministration with ICI182,780, the decreases in the immobility time in the FST and TST caused by estradiol and TPLB were significantly attenuated.

### 3.6. Effects of Estradiol and TPLB on Cognitive Performance after Coadministration with ICI182,780

In the training trials, the latency of OVX mice treated with both estradiol and TPLB was pronouncedly shorter than that of those treated with vehicle at Day 4 through Day 6. The OVX mice coadministrated with estradiol and ICI182,780 showed no significant changes on the latency to the platform compared to those treated with estradiol only. However, cotreatment with TPLB and ICI182,780 induced significantly longer latency to the platform than with TPLB treatment alone at Day 2 through Day 6.

In the probe trial, the OVX mice treated with estradiol and TPLB had more duration in and shorter latency to the target zone than those treated with vehicle (Figures 3(o)–3(r)). After coadministration with ICI182,780, the OVX mice showed no significant changes on the duration in, entry number into, and latency to the target zone compared to those treated with estradiol or TPLB alone. The representative navigation paths of the five groups are shown in Figures 3(i)–3(n).

### 3.7. Effects of Estradiol and TPLB on Brain Regional and Uterine Estrogen Receptors after Coadministration with ICI182,780

Significant group effects were observed on the expression of both ER*α* and ER*β* in all the three brain regions and uterus examined (Figures 2(a)–2(h)). Except for cortical ER*β*, estradiol treatment completely reversed the OVX-induced decreases of ER*α* and ER*β* in the four tissues examined. In addition to cortical ER*α*, TPLB treatment completely reversed the OVX-induced decreases of ER*β* in the three brain regions. After coadministration with ICI, the treated OVX mice showed significant decreases in the expression levels of the uterine ER*α* and ER*β*, hypothalamic ER*β*, and hippocampal ER*α* and ER*β* compared to those treated with estradiol. Interestingly, cotreatment with TPLB and ICI182,780 induced remarkable decreases in the ER*β* expression level in the three brain regions examined compared to the TPLB-treated OVX mice. Additionally, the OVX mice cotreated with TPLB and ICI182,780 also showed a remarkable decrease in the hippocampal ER*α* expression level compared to the TPLB-treated OVX mice.

### 3.8. TPLB Binding to ERs

To further explore the effects of TPLB on ERs, we examined whether TPLB could directly bind to ERs in vitro. As shown in Figures 2(i) and 2(j), estradiol has a potent binding ability to ER *α* and *β* (*P* = 0.0001). The binding ability of TPLB to ER*β* is greater than TPLB to ER*α*. Only 600 *μ*g/mL TPLB had significantly lower fluorescence polarization than Fluormone ligand when competed for ER*α* binding, while TPLB showed significantly competitive binding ability to ER*β* at the concentration of 66.7 *μ*g/mL.

### 3.9. Effects of Estradiol and TPLB on Brain Regional and Uterine Neurotrophins after Coadministration with ICI182,780

Significant group differences were also observed on GDNF, NGF, and BDNF in the prefrontal cortex, hippocampus, and uterus (Figure 4). Estradiol treatment almost completely reversed the OVX-induced decreases of the three neurotrophins in the three tissues except for hippocampal NGF and cortical GDNF. Except for uterine NGF, TPLB treatment completely reversed the OVX-induced decreases of the three neurotrophins in the three tissues. The OVX mice cotreated with estradiol and ICI182,780 showed significant decreases in the expression levels of BDNF in the three tissues, hippocampal GDNF, uterine, and prefrontal NGF compared to those treated with estradiol alone. Coadministration with TPLB and ICI182,780 induced widespread decreases in the BDNF, GDNF, and NGF expression levels in the three tissues except for uterine NGF compared to TPLB treatment alone.

### 3.10. Effects of Estradiol and TPLB on GABAergic and Glutamatergic Systems after Coadministration with ICI182,780

Gamma-aminobutyric acid (GABA) is the principle inhibitory neurotransmitter, while glutamate is the principle excitatory neurotransmitter in the central nervous system (CNS). The equilibrium of the two system plays an important role in neuronal excitability, synaptic plasticity, and cognitive functions [28]. OVX induced a dramatic decrease in GABA levels but a remarkable increase in glutamate levels in the brain regions, which were completely reversed by both estradiol and TPLB treatment (Figures 5(a)–5(c), Figures 6(a) and 6(b)). After cotreatment with ICI182,780, the OVX mice showed a significant decrease in the GABA level and a significant increase in the glutamate level in the three brain regions compared to those treated with estradiol or TPLB alone.

GABA_A_ receptors play an important role in modulating memory acquisition, and the *α*_1_ subunit-containing GABA_A_ is the major subtype accounting for about 60% of all GABA_A_ receptors in the brain [29]. GAD67 catalyzes the process of GABA synthesis for synaptic transmission [30]. In this experiment, estradiol and TPLB treatment completely reversed the OVX-induced decreases in the two biomarkers of the GABAergic system, GABA_A*α*1_ and GAD67, in the hypothalamus, hippocampus, and prefrontal cortex (Figures 5(d)–5(i)). The OVX mice coadministrated with estradiol and ICI182,780 showed significant decreases in the expression levels in GAD67 and GABA_A*α*1_ in the three brain regions except for cortical GABA_A*α*1_ compared to those treated with estradiol alone. Cotreatment with TPLB and ICI182,780 induced significant decreases in the expression levels of hypothalamic and hippocampal GAD67 and hippocampal and cortical GABA_A*α*1_ compared with TPLB treatment alone.

In addition, NMDA receptors are also thought to be involved in the estrogen-enhancing effect on spatial reference memory [31]. NMDAR1 mediates neuronal functions in glutamate neurotransmission, and the activation of NMDARs induces additional Ca^2+^ to enter the cell and further activates Ca^2+^-dependent signaling pathways [32]. In the current study, estradiol and TPLB treatment completely reversed the OVX-induced changes in the phosphorylation of CaMKII and NMDAR1 levels in the hippocampus and prefrontal cortex except for cortical NMDAR1 (Figures 6(c)–6(f)). The OVX mice cotreated with TPLB and ICI182,780 showed a significant decrease in the p-CaMKII/CaMKII ratio and a significant increase in the NMDAR1 expression level compared to those treated with TPLB alone. However, coadministration with estradiol and ICI182,780 caused no significant changes on the p-CaMKII/CaMKII ratio and NMDAR1 expression levels compared to those only treated with estradiol.

### 3.11. Effects of Estradiol and TPLB on the Serotoninergic and Catecholaminergic Systems after Coadministration with ICI182,780

Serotonin transporter (ST) transports serotonin from the synaptic cleft back to the presynaptic neuron and recycles it, thus playing a key role in regulating serotonergic neurotransmission [33]. Tyrosine hydroxylase (TH) is a rate-limiting enzyme of catecholamine biosynthesis, and brain catecholaminergic neuronal systems play a crucial role in the development of central neuronal regulatory circuits [34]. Estradiol and TPLB treatment completely reversed the OVX-induced decreases in ST and TH in the hippocampus and prefrontal cortex (Figure 7). After coadministration with ICI182,780, the OVX mice showed significant decreases in the ST and TH expression levels in the two regions compared to those treated with estradiol or TPLB alone. Additionally, the OVX mice cotreated with TPLB and ICI182,780 also showed a significant decrease in the hypothalamic ST expression level compared to those treated with TPLB alone.

### 3.12. Bioinformatic Analysis

Principal component analysis (PCA) and hierarchical cluster analysis (HCA) were used for clustering analysis. PCA and HCA plots (Figure 8) revealed that samples were clearly classified into four categories, including sham, OVX, E_2_, and TPLB.

**Figure 9:**
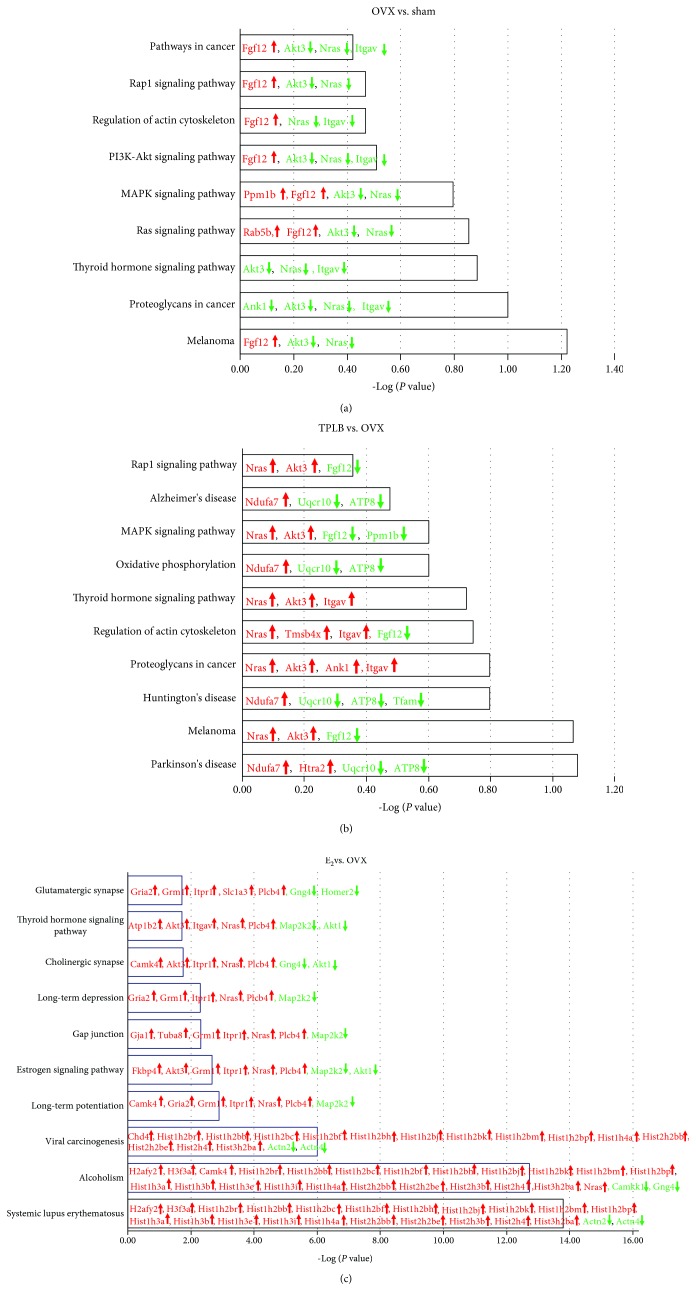
KEGG pathway analysis of three comparison groups: OVX vs. sham (a), TPLB vs. OVX (b), and E_2_ vs. OVX (c). The *X*-axis displayed the negative log of the *P* value calculated by the right-tailed Fisher exact test, while *Y*-axis represented the involved pathway. Proteins in green letters were downregulated, and those in red were upregulated.

Of the 2723 identified proteins, 102 proteins differentially expressed in OVX and Sham, 257 in E_2_ and OVX, and 119 in TPLB and OVX groups were submitted for GO functional annotation and analysis. The top ten significantly enriched GO terms for three comparison groups are presented in S-Fig.  9 according to *P* value and number of genes. E_2_ treatment had large influences on the functions of structural constituent of ribosome, poly(A) RNA binding and rRNA binding, and the processes of nucleosome assembly, translation, and DNA methylation on cytosine on nuclear nucleosome, indicating the involvement of both genetic and epigenetic effects. TPLB had particular effects on the functions of oxygen binding and peptide-methionine (S)-S-oxide reductase activity in the mitochondrion.

KEGG pathway database was used to analyze major biological pathways and relevant regulatory processes involving differentially expressed proteins. KEGG pathway database is a complex database system that consists of a collection of manually drawn pathway maps that represent the updated knowledge on the molecular interaction, reaction, and relation networks for metabolism, genetic information processing, environmental information processing, cellular processes, organismal systems, human diseases, and drug development [35]. The top ten pathways for three comparison groups are present in Figure 9 and can be found on the website https://www.genome.jp/kegg/pathway.html. The involved gene names and the corresponding protein names were described in S-Table 2. Although the involved pathways in OVX versus sham and TPLB versus OVX groups were not significantly enriched (*P* ≥ 0.06 and *P* ≥ 0.083, respectively), the results revealed that TPLB treatment completely reversed the OVX-induced upregulation of proteins with gene names of Fgf12 and Ppm1b, as well as downregulation of proteins with gene names of Akt3, Nras, Ank1, and Itgav. Multiple pathways involved in neurodegenerative diseases and oxidative phosphorylation were enriched in TPLB versus OVX groups, with the regulation of several mitochondrial proteins with gene names of Ndufa7, Uqcr10, and ATP8. In E_2_ versus OVX groups, the top three enriched pathways were systemic lupus erythematosus, alcoholism, and viral carcinogenesis, which were closely associated with the upregulation of Histone 2A, 2B, 3, and 4. Long-term potentiation and estrogen receptor signaling were also significantly enriched, with the upregulation of proteins with gene names of Grm1, Itpr1, PLC, Akt3, and Nras, together with the downregulation of dual specificity mitogen-activated protein kinase kinase 2.

**Figure 10:**
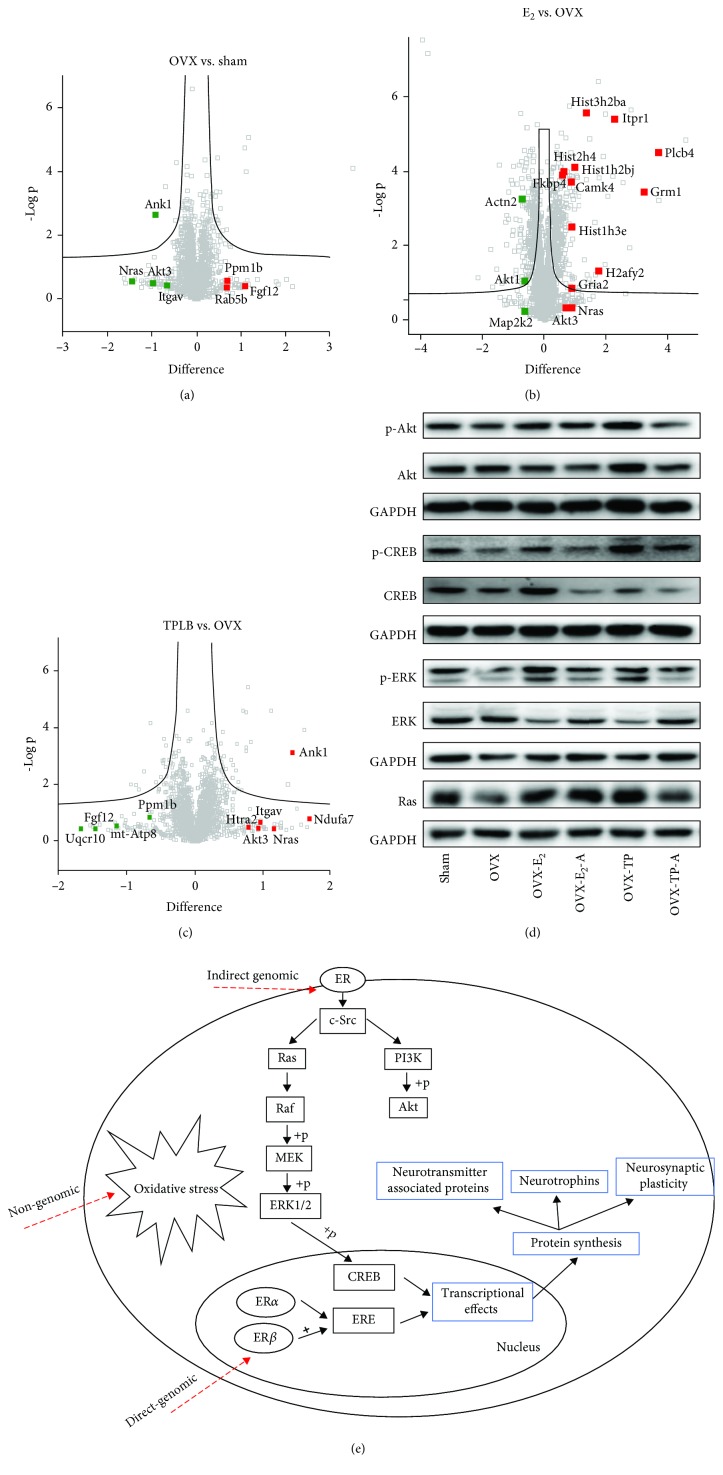
Volcano plot and validation of proteomics results and a diagrammatic illustration summarizing the potential mechanisms of TPLB. Volcano plots of three comparison groups: OVX vs. sham (a), TPLB vs. OVX (b), and E_2_ vs. OVX (c) with red representing upregulated proteins and green indicating downregulated proteins. (d) Representative images indicating the expression levels of Ras, p-Akt, Akt, p-ERK, ERK, p-CREB, and CREB in the prefrontal cortex; (e) a diagrammatic illustration summarizing the potential mechanisms of TPLB on the menopause-associated psychiatric disorders.

The changes of several crucial genes were marked in volcano plot (Figure 10). The shared mechanism between TPLB and estradiol was mediated by Nras and Akt3 in the estrogen receptor pathway, which was validated by western blotting (Figure 10(d)). OVX downregulated Ras, which plays a crucial role in cellular signaling transduction. OVX also reduced the phosphorylation of protein kinase B (Akt) and extracellular signal-regulated kinase (ERK), thus inducing the decreased phosphorylation of cyclic AMP-response element-binding protein (CREB). Estradiol and TPLB significantly upregulated the expression of these proteins, which, however, was almost completely blocked by ICI182,780.

## 4. Discussion

This study showed that the exposure of OVX mice to chronic stress caused various aberrant behaviors indicative of anxiety, depression, and cognitive deterioration in multiple behavioral test paradigms. Consistent with our previous study [8], this study revealed that TPLB and estradiol almost completely reversed these aberrant behaviors, indicating the comparable efficacy of both agents in improving menopause-related psychiatric symptoms. The addition of the ER antagonist ICI182,780, however, largely attenuated anxiolytic and antidepressant effects of both agents and the cognition-improving effects of TPLB during the acquisition trials in the water maze test but did not have significant effects on the nootropic effects of estradiol and the effects of TPLB in improving cognitive performance in the probe trial. In the water maze test, the spatial learning ability and spatial memory are examined in the training trials and the probe trial, respectively [36, 37]. These results suggested that, while both agents share similar mechanisms responsible for their anxiolytic and antidepressant effects, they may have distinct mechanisms associated with their nootropic effects. The effects of TPLB on spatial learning seem to be ER-dependent, whereas its effects on spatial memory, like estradiol, may be related to the modulation of membrane-associated G protein-coupled estrogen receptor 1 (GPR30), which is heavily involved in the estrogenic mediation of learning and memory via rapid signaling mechanisms [38].

Although ER*α* and ER*β*, the two classical estrogen receptor subtypes, are widely involved in the pathophysiology of menopause-associated metabolic, neurological, and psychiatric disorders, they have distinct anatomical distribution patterns and differential physiological processes in the brain and peripheral organs and even counteract each other [39–42]. While ER*α* is more closely associated with cognitive impairment, as demonstrated in OVX animals and postmenopausal women, ER*β* plays a predominant role in the pathogenesis of depression- and anxiety-like behavior [43–47]. One recent study has shown that activation of both ER*α* and ER*β* restored OVX-induced recognition memory deficit at earlier stages of consolidation [48]. In addition to ER*α* and ER*β*, GPR30 also has been confirmed to have an important role in cholinergic function and synaptic plasticity by phosphorylating the classical intracellular ER*α*, functioning as ER*α* collaborator [49–51]. The current study found that OVX caused widespread suppression in the expression levels of the two receptors in the uterus and brain regions. Chronic estradiol treatment, however, completely restored the expression levels of the two receptors in the uterus, hypothalamus, and hippocampus and ER*α* in the prefrontal cortex. Likewise, chronic TPLB also reversed the OVX-induced decrease of the prefrontal ER*α* expression and enhanced the expression levels of ER*β* in the uterus and three brain regions examined. Nevertheless, ICI182,780 only blocked TPLB-induced upregulation of ER*β* in multiple brain regions but had no significant effects on prefrontal ER*α*. On the other hand, in vitro binding experiment revealed that estradiol had similar potencies in binding affinity for ER*α* and ER*β*, but TPLB displayed a higher affinity at ER*β* than ER*α*. These results are in line with the behavioral findings of this study, suggesting that the differential behavioral effects of EPLB and estrogen therapy may be associated with their differences in modulating the two ER subtypes.

It is well documented that neurotrophic changes are closely associated with menopause-related anxiety, depression, and cognitive deterioration [52, 53]. NGF, GDNF, and BDNF are the three most abundant neurotrophins existing in the adult brain [54]. These neurotrophins are also highly expressed in the female reproductive system and exert their biological roles in uterine growth and proliferation [55, 56]. In this study, we found that OVX caused a widespread decrease in the expression levels of the three neurotrophins in the prefrontal cortex, hippocampus, and uterus. Both estradiol and TPLB consistently reversed the OVX-induced decrease of the BDNF expression in the three tissues examined. Furthermore, TPLB had significant effects in restoring the expression of hippocampal NGF and prefrontal GDNF without influencing uterine NGF. In contrast, estradiol significantly reversed the OVX-induced decrease of uterine NGF, but it had no significant effects on hippocampal NGF and prefrontal GDNF. A high level of peripheral NGF has been shown to be associated with ovarian and breast cancer, polycystic ovarian syndrome (PCOS), and endometriosis [57, 58], providing evidence for the increased risk of breast and endometrial cancer caused by long-term estrogen replacement therapy [59, 60]. It also suggests that TPLB may have a better safety profile than estradiol therapy as the former agent has no effects on the uterine NGF expression. Interestingly, the TPLB-induced upregulation of neurotrophins was completely blocked by ICI182,780, indicating that the neurotrophic effects of TPLB were mainly mediated by ERs.

The beneficial effects of estradiol on menopause-associated psychiatric symptoms are closely associated with multiple brain neurotransmitters, including serotoninergic, catecholaminergic, GABAergic, and glutamatergic neurons, and their receptors [61–64]. Low GABAergic activity may play a key role in the pathophysiology of mood disorders [65]. The GABAergic system has been verified to be related to the cognitive dysfunction in OVX animals [66]. Meanwhile, the role of the glutamatergic system in the neurobiology and treatment of mood disorders has attracted increasing attention [67, 68]. This is possibly attributed to the activation of intracellular kinases and phosphatases caused by calcium influx through NMDAR ion channels, thereby altering the characteristics of the synapse and providing the basis for neuronal transmission [69]. Changes in the serotoninergic system have been linked with mood, anxiety, and cognitive disorders [70]. Interactions between estrogen and serotonin have long been acknowledged with regard to mood and cognition, and estrogen induces changes in serotonin transmission, binding, and metabolism in the brain regions [71]. Catecholaminergic neurons also play important roles in diverse cognitive, motor, and endocrine functions, thus participating in multiple psychiatric and neurodegenerative disorders [72]. Consistent with the previous reports [30, 73], this study revealed that OVX induced significant decreases in the GABA levels, the GAD67 and GABA_A*α*1_ expression in the GABAergic system, and the TH expression in the dopaminergic system in the hypothalamus, hippocampus, and prefrontal cortex. Estradiol and TPLB completely restored and even enhanced the GABA levels, GAD67 and GABA_A*α*1_, and ST and TH expression levels in the three brain regions. Furthermore, the effects of TPLB on the serotoninergic, catecholaminergic, and GABAergic systems were also almost blocked by ICI182,780, confirming the mediation of ERs. This study revealed that estradiol and TPLB decreased the glutamate levels and NMDAR1 expression and elevated the p-CaMKII/CaMKII ratio in the hippocampus and prefrontal cortex. Similarly, the effects of TPLB on the glutamatergic system and synaptic plasticity were also ER-dependent because ICI182,780 completely blocked the effects.

Proteomics is a systematic approach for conducting protein studies on a large-scale, providing substantial information about protein abundance, modification, and interactions [74]. In this study, we found that estradiol and TPLB shared the same mechanisms via the upregulation of Ras/Akt/ERK/CREB signaling in the estrogen receptor pathway (Figure 10(e)), which plays an important role in the neurodegenerative diseases and neuron survival [75–77]. Furthermore, E_2_ had a major effect on the epigenetics, causing DNA methylation and histone modifications. The results were in agreement with the previous study [78]. However, a growing number of studies revealed that epigenetics also mediated the regulation of estrogen signaling in breast cancer [79–81]. One clinical study has shown that endogenous estrogen exposure was associated with repetitive element DNA methylation in healthy postmenopausal women, which may help to explain why estrogen exposure impacts cancer risk [82], which was supported by another clinical study that has indicated that DNA methylation at cytosine-phosphate-guanine islands had a significant association with breast cancer susceptibility [83]. Unlike E_2_, TPLB predominately influenced the mitochondrial oxidative stress. A substantial body of evidence has revealed the presence of oxidative stress in menopausal transition [84, 85] and confirmed the involvement of oxidative stress in the development of OVX-induced pathophysiological changes [86]. Oxidative stress can contribute to the pathogenesis of menopause-related disturbances and diseases [87]. Moreover, some studies have demonstrated that regulation of oxidative stress resulted in the attenuation of cognitive deficits [88], suggesting that the antimenopausal effects of TPLB might be mediated via balance of oxidative stress.

Several limitations of this study should be noted. Some results of proteomic analysis were not further validated in the current study, and we will focus on it in our future study. Furthermore, behavioral, neurochemical, and molecular methodologies used in this study could not directly detect functional changes at subcellular and molecular level. This may limit our explanation to the findings. Electrophysiological and advanced pharmacological approaches should be applied in the future to examine the exact binding process of TPLB to the estrogen receptor subtypes.

In summary, the antimenopausal effects of TPLB with a better safety profile seem to be achieved via multiple mechanisms distinct from estrogen therapy. First, the effects of TPLB were closely associated with the predominant activation of estrogen receptors, along with the regulation of neurotransmitters and neurotrophins in the brain, but with minor effects on the peripheral organs. Moreover, the effects of TPLB on the synaptic transmission and neurotrophins were mainly mediated by activation of ERs. Second, E_2_ had similar potencies in binding affinity for ER*α* and ER*β*, thus causing widespread genetic and epigenetic effects, whereas TPLB displayed a higher affinity at ER*β* than ER*α*, which may trigger the specific Ras/Akt/ERK/CREB signaling without affecting any epigenetic activity. Lastly, TPLB additionally influenced the mitochondrial oxidative stress. We suggest that TPLB could serve as a novel agent for menopause syndrome.

## Data Availability

The authors confirm that the data supporting the findings of this study are available within the article and its supplementary materials.

## Abbreviations

Akt: Protein kinase B
ANCOVA: Analysis of covariance
ANOVA: One-way analysis of variance
BDNF: Brain-derived neurotrophic factor
CaMKII: Calmodulin-dependent protein kinase II
CREB: cAMP-response element-binding protein
CUMS: Chronic unpredictable mild stress
E_2_: Estradiol
ELISA: Enzyme-linked immunosorbent assay
ERK: Extracellular signal-regulated kinases
ER*α* and ER*β*: Estrogen receptors alpha and beta
FASP: Filter-aided sample preparation
FST: Forced swimming test
GABA_A*α*1_: *α*-Aminobutyric acid A receptor alpha 1 subunit
GAD67: Glutamate decarboxylase 67
GAPDH: Glyceraldehyde-3-phosphate dehydrogenase
GDNF: Glial cell-derived neurotrophic factor
GO: Gene ontology
HCA: Hierarchical cluster analysis
HPLC: High-performance liquid chromatography
MWM: Morris water maze
NGF: Nerve growth factor
NMDAR1: N-Methyl-D-aspartate receptor subunit 1
OFT: Open-field test
EPM: Elevated plus maze
OVX: Ovariectomized
PCA: Principal component analysis
SEM: Standard error of the mean
SPT: Sucrose preference test
ST: Serotonin transporter
TH: Tyrosine hydroxylase
TPLB: Total polysaccharides of lily bulb
TST: Tail suspension test.

## Supplementary Materials

Supplementary Materials S-Table 1: list of various antibodies used in western blot analysis. S-Table 2: the involved gene names and the corresponding protein names in the KEGG pathways and volcano plots. S-Fig. 1: the effects of three doses of TPLB on body weight, uterine weight, and serum estradiol. (A) Variation of net body weight on the 14th day after OVX; (B) ratio of the uterus to body weight at the end of the 5-week treatment period; (C) serum estradiol levels. Data are expressed as mean ± SEM (*n* = 10) and examined with one-way ANOVA, followed by post hoc Dunnett's test: ^∗^ *P* < 0.05, ^∗∗^ *P* < 0.01, and ^∗∗∗^ *P* < 0.001 vs. the sham group; ^#^ *P* < 0.05, ^##^ *P* < 0.01, and ^###^ *P* < 0.001 vs. the OVX group. S-Fig. 2: the representational HPLC-DAD chromatograms for the measurement of glutamate and GABA (A) in the hypothalamus (B), hippocampus (C), and prefrontal cortex (D). S-Fig. 3: the effects of TPLB on the GABAergic system in the brain regions. GABA levels (*n* = 6) in the hypothalamus (A), hippocampus (B), and prefrontal cortex (C); representative images indicating the expression levels (*n* = 3) of GABA_A*α*1_ and GAD67 and their quantification analysis in the hypothalamus (D-E), hippocampus (F-G), and prefrontal cortex (H-I). Data are expressed as mean ± SEM and examined with one-way ANOVA, followed by post hoc Dunnett's test: ^∗^ *P* < 0.05, ^∗∗^ *P* < 0.01, and ^∗∗∗^ *P* < 0.001 vs. the sham group; ^#^ *P* < 0.05, ^##^ *P* < 0.01, and ^###^ *P* < 0.001 vs. the OVX group. S-Fig. 4: the effects of TPLB on the glutamatergic system in the brain regions. Glutamate levels (*n* = 6) in the hippocampus (A) and prefrontal cortex (B); representative images indicating the expression levels (*n* = 3) of NMDAR1 and CaMKII and their quantification analysis in the hippocampus (C-D) and prefrontal cortex (E-F). Data are expressed as mean ± SEM and examined with one-way ANOVA, followed by post hoc Dunnett's test: ^∗^ *P* < 0.05, ^∗∗^ *P* < 0.01, and ^∗∗∗^ *P* < 0.001; ^#^ *P* < 0.05, ^##^ *P* < 0.01, and ^###^ *P* < 0.001 vs. the OVX group. S-Fig. 5: the effects of three doses of TPLB on the serotonergic and catecholaminergic systems in the brain regions. Representative images indicating the expression levels of ST and SR-1A, TH, and their quantification analysis in the hypothalamus (A-B), hippocampus (C-D), and prefrontal cortex (E-F). Data are expressed as mean ± SEM (*n* = 3) and examined with one-way ANOVA, followed by post hoc Dunnett's test: ^∗^ *P* < 0.05, ^∗∗^ *P* < 0.01, and ^∗∗∗^ *P* < 0.001; ^#^ *P* < 0.05, ^##^ *P* < 0.01, and ^###^ *P* < 0.001 vs. the OVX group. S-Fig. 6: the effects of three doses of TPLB on the expression levels of neurotrophins, BDNF, GDNF, and NGF, in the uterus, hippocampus, and prefrontal cortex. Representative images indicating the expression levels of three neurotrophins and their quantification analysis in the uterus (A-B), hippocampus (CD), and prefrontal cortex (E-F). Data are expressed as mean ± SEM (*n* = 3) and examined with one-way ANOVA, followed by post hoc Dunnett's test: ^∗^ *P* < 0.05, ^∗∗^ *P* < 0.01, and ^∗∗∗^ *P* < 0.001; ^#^ *P* < 0.05, ^##^ *P* < 0.01, and ^###^ *P* < 0.001 vs. the OVX group. S-Fig. 7: the effects of three doses of TPLB on the expression levels of estrogen receptors, ER*α* and ER*β*, in the uterus and brain regions. Representative images indicating the expression levels of ER*α* and ER*β* and their quantification analysis in the uterus (A-B), hypothalamus (C-D), hippocampus (E-F), and prefrontal cortex (G-H). Data are expressed as mean ± SEM (*n* = 3) and examined with one-way ANOVA, followed by post hoc Dunnett's test: ^∗^ *P* < 0.05, ^∗∗^ *P* < 0.01, and ^∗∗∗^ *P* < 0.001; ^#^ *P* < 0.05, ^##^ *P* < 0.01, and ^###^ *P* < 0.001 vs. the OVX group. S-Fig. 8: the effects of estradiol and TPLB on body weight, uterine weight, and serum estradiol after coadministration with ICI182,780. (A) Variation of net body weight on the 14th day after OVX; (B) ratio of the uterus to body weight at the end of the 5-week treatment period; (C) serum estradiol levels. Data are expressed as mean ± SEM (*n* = 10) and examined with one-way ANOVA, followed by post hoc Sidak's multiple comparisons test: ^∗^ *P* < 0.05, ^∗∗^ *P* < 0.01, and ^∗∗∗^ *P* < 0.001 vs. the sham group; ^#^ *P* < 0.05, ^##^ *P* < 0.01, and ^###^ *P* < 0.001 vs. the OVX group; ^&^ *P* < 0.05, ^&&^ *P* < 0.01, and ^&&&^ *P* < 0.001 vs. the E2 group or TP group. S-Fig. 9: the ten most significantly enriched three terms in gene ontology (GO): biological process, cell component, and molecular function.

## References

[1] Perich T., Ussher J., Meade T. (2017). Menopause and illness course in bipolar disorder: a systematic review. *Bipolar Disorders*.

[2] Koebele S. V., Bimonte-Nelson H. A. (2017). The endocrine-brain-aging triad where many paths meet: female reproductive hormone changes at midlife and their influence on circuits important for learning and memory. *Experimental Gerontology*.

[3] Jenabi E., Shobeiri F., Hazavehei S. M. M., Roshanaei G. (2015). Assessment of questionnaire measuring quality of life in menopausal women: a systematic review. *Oman Medical Journal*.

[4] Lobo R. A. (2017). Hormone-replacement therapy: current thinking. *Nature Reviews Endocrinology*.

[5] Fernandez E., Gallus S., Bosetti C., Franceschi S., Negri E., La Vecchia C. (2003). Hormone replacement therapy and cancer risk: a systematic analysis from a network of case-control studies. *International Journal of Cancer*.

[6] Collaborative Group on Epidemiological Studies of Ovarian Cancer (2015). Menopausal hormone use and ovarian cancer risk: individual participant meta-analysis of 52 epidemiological studies. *The Lancet*.

[7] Tonob D., Melby M. K. (2017). Broadening our perspectives on complementary and alternative medicine for menopause: a narrative review. *Maturitas*.

[8] Zhou X.-D., Shi D.-D., Wang H.-N., Tan Q.-R., Zhang Z.-J. (2019). Aqueous extract of lily bulb ameliorates menopause-like behavior in ovariectomized mice with novel brain-uterus mechanisms distinct from estrogen therapy. *Biomedicine & Pharmacotherapy*.

[9] Xu Z., Wang H., Wang B. (2016). Characterization and antioxidant activities of polysaccharides from the leaves of *Lilium lancifolium* Thunb. *International Journal of Biological Macromolecules*.

[10] Gao J., Zhang T., Jin Z. Y. (2015). Structural characterisation, physicochemical properties and antioxidant activity of polysaccharide from *Lilium lancifolium* Thunb. *Food Chemistry*.

[11] Pan G., Xie Z., Huang S. (2017). Immune-enhancing effects of polysaccharides extracted from *Lilium lancifolium* Thunb. *International Immunopharmacology*.

[12] Chen Z. G., Zhang D. N., Zhu Q., Yang Q. H., Han Y. B. (2014). Purification, preliminary characterization and in vitro immunomodulatory activity of tiger lily polysaccharide. *Carbohydrate Polymers*.

[13] Sun X., Gao R. L., Xiong Y. K., Huang Q. C., Xu M. (2014). Antitumor and immunomodulatory effects of a water-soluble polysaccharide from *Lilii Bulbus* in mice. *Carbohydrate Polymers*.

[14] Han H., Xie H. (2013). A study on the extraction and purification process of lily polysaccharide and its anti-tumor effect. *African Journal of Traditional, Complementary and Alternative Medicines*.

[15] Zhang T., Gao J., Jin Z. Y., Xu X. M., Chen H. Q. (2014). Protective effects of polysaccharides from *Lilium lancifolium* on streptozotocin-induced diabetic mice. *International Journal of Biological Macromolecules*.

[16] Bean L. A., Ianov L., Foster T. C. (2014). Estrogen receptors, the hippocampus, and memory. *The Neuroscientist*.

[17] Zhou X.-D., Shi D. D., Zhang Z. J. (2019). Antidepressant and anxiolytic effects of the proprietary Chinese medicine Shexiang Baoxin pill in mice with chronic unpredictable mild stress. *Journal of Food and Drug Analysis*.

[18] Zha S., Zhao Q., Chen J. (2014). Extraction, purification and antioxidant activities of the polysaccharides from maca (*Lepidium meyenii*). *Carbohydrate Polymers*.

[19] Saha A. K., Brewer C. F. (1994). Determination of the concentrations of oligosaccharides, complex type carbohydrates, and glycoproteins using the phenol-sulfuric acid method. *Carbohydrate Research*.

[20] Gresack J. E., Frick K. M. (2004). Environmental enrichment reduces the mnemonic and neural benefits of estrogen. *Neuroscience*.

[21] Gresack J. E., Frick K. M. (2006). Post-training estrogen enhances spatial and object memory consolidation in female mice. *Pharmacology Biochemistry and Behavior*.

[22] Jung Koo H., Sohn E.-H., Kim Y.-J., Jang S.-A., Namkoong S., Chan Kang S. (2014). Effect of the combinatory mixture of *Rubus coreanus* Miquel and *Astragalus membranaceus* Bunge extracts on ovariectomy-induced osteoporosis in mice and anti-RANK signaling effect. *Journal of Ethnopharmacology*.

[23] Shi D. D., Dong C. M., Ho L. C. (2018). Resveratrol, a natural polyphenol, prevents chemotherapy-induced cognitive impairment: involvement of cytokine modulation and neuroprotection. *Neurobiology of Disease*.

[24] Huo T., Chang B., Zhang Y., Chen Z., Li W., Jiang H. (2012). Alteration of amino acid neurotransmitters in brain tissues of immature rats treated with realgar. *Journal of Pharmaceutical and Biomedical Analysis*.

[25] Wisniewski J. R., Zougman A., Nagaraj N., Mann M. (2009). Universal sample preparation method for proteome analysis. *Nature Methods*.

[26] Chan L. H., Zhou L., Ng K. Y. (2018). PRMT6 regulates RAS/RAF binding and MEK/ERK-mediated cancer stemness activities in hepatocellular carcinoma through CRAF methylation. *Cell Reports*.

[27] Mi H., Muruganujan A., Thomas P. D. (2013). PANTHER in 2013: modeling the evolution of gene function, and other gene attributes, in the context of phylogenetic trees. *Nucleic Acids Research*.

[28] Samardzic J., Jadzic D., Hencic B., Jancic J., Strac D. S. (2018). Introductory Chapter: GABA/Glutamate Balance: A Key for Normal Brain Functioning. *GABA And Glutamate - New Developments In Neurotransmission Research*.

[29] Shojaee A., Taherianfard M. (2017). Effects of gonadectomy and avoidance learning on the GABA_A*α*1_ receptor density in the prefrontal cortex of male and female rats. *Neurophysiology*.

[30] Nakamura N. H., Rosell D. R., Akama K. T., McEwen B. S. (2004). Estrogen and ovariectomy regulate mRNA and protein of glutamic acid decarboxylases and cation-chloride cotransporters in the adult rat hippocampus. *Neuroendocrinology*.

[31] el-Bakri N. K., Islam A., Zhu S. (2004). Effects of estrogen and progesterone treatment on rat hippocampal NMDA receptors: relationship to Morris water maze performance. *Journal of Cellular and Molecular Medicine*.

[32] Zhao B., Wang Y., Li Y. (2015). Differential phosphorylation of NMDAR1–CaMKII–MAPKs in the rat nucleus accumbens following chronic ethanol exposure. *Neuroscience Letters*.

[33] Jovanovic H., Kocoska-Maras L., Radestad A. F. (2015). Effects of estrogen and testosterone treatment on serotonin transporter binding in the brain of surgically postmenopausal women – a PET study. *NeuroImage*.

[34] Kvetnansky R., Sabban E. L., Palkovits M. (2009). Catecholaminergic systems in stress: structural and molecular genetic approaches. *Physiological Reviews*.

[35] Kanehisa M., Furumichi M., Tanabe M., Sato Y., Morishima K. (2017). KEGG: new perspectives on genomes, pathways, diseases and drugs. *Nucleic Acids Research*.

[36] Poulose S. M., Rabin B. M., Bielinski D. F. (2017). Neurochemical differences in learning and memory paradigms among rats supplemented with anthocyanin-rich blueberry diets and exposed to acute doses of ^56^Fe particles. *Life Sciences in Space Research*.

[37] Barnhart C. D., Yang D., Lein P. J. (2015). Using the Morris water maze to assess spatial learning and memory in weanling mice. *PLoS One*.

[38] Lymer J., Robinson A., Winters B. D., Choleris E. (2017). Rapid effects of dorsal hippocampal G-protein coupled estrogen receptor on learning in female mice. *Psychoneuroendocrinology*.

[39] Crider A., Pillai A. (2017). Estrogen signaling as a therapeutic target in neurodevelopmental disorders. *The Journal of Pharmacology and Experimental Therapeutics*.

[40] Deroo B. J., Korach K. S. (2006). Estrogen receptors and human disease. *The Journal of Clinical Investigation*.

[41] Vargas K. G., Milic J., Zaciragic A. (2016). The functions of estrogen receptor beta in the female brain: a systematic review. *Maturitas*.

[42] Morani A., Warner M., Gustafsson J. A. (2008). Biological functions and clinical implications of oestrogen receptors alfa and beta in epithelial tissues. *Journal of Internal Medicine*.

[43] Xu Y., Sheng H., Bao Q., Wang Y., Lu J., Ni X. (2016). NLRP3 inflammasome activation mediates estrogen deficiency-induced depression- and anxiety-like behavior and hippocampal inflammation in mice. *Brain, Behavior, and Immunity*.

[44] Pereira L. M., Bastos C. P., de Souza J. M., Ribeiro F. M., Pereira G. S. (2014). Estradiol enhances object recognition memory in Swiss female mice by activating hippocampal estrogen receptor *α*. *Neurobiology of Learning and Memory*.

[45] Olsen L., Rasmussen H. B., Hansen T. (2006). Estrogen receptor alpha and risk for cognitive impairment in postmenopausal women. *Psychiatric Genetics*.

[46] Sundermann E. E., Maki P. M., Bishop J. R. (2010). A review of estrogen receptor *α* gene (*ESR1*) polymorphisms, mood, and cognition. *Menopause*.

[47] Jacome L. F., Gautreaux C., Inagaki T. (2010). Estradiol and ER*β* agonists enhance recognition memory, and DPN, an ER*β* agonist, alters brain monoamines. *Neurobiology of Learning and Memory*.

[48] Bastos C. P., Pereira L. M., Ferreira-Vieira T. H. (2015). Object recognition memory deficit and depressive-like behavior caused by chronic ovariectomy can be transitorialy recovered by the acute activation of hippocampal estrogen receptors. *Psychoneuroendocrinology*.

[49] Xu W., Cao J., Zhou Y., Wang L., Zhu G. (2018). GPR30 activation improves memory and facilitates DHPG-induced LTD in the hippocampal CA3 of middle-aged mice. *Neurobiology of Learning and Memory*.

[50] Hawley W. R., Grissom E. M., Moody N. M., Dohanich G. P., Vasudevan N. (2014). Activation of G-protein-coupled receptor 30 is sufficient to enhance spatial recognition memory in ovariectomized rats. *Behavioural Brain Research*.

[51] Hadjimarkou M. M., Vasudevan N. (2018). GPER1/GPR30 in the brain: crosstalk with classical estrogen receptors and implications for behavior. *The Journal of Steroid Biochemistry and Molecular Biology*.

[52] Engler-Chiurazzi E., Tsang C., Nonnenmacher S. (2011). Tonic Premarin dose-dependently enhances memory, affects neurotrophin protein levels and alters gene expression in middle-aged rats. *Neurobiology of Aging*.

[53] Van Kempen T. A., Gorecka J., Gonzalez A. D., Soeda F., Milner T. A., Waters E. M. (2014). Characterization of neural estrogen signaling and neurotrophic changes in the accelerated ovarian failure mouse model of menopause. *Endocrinology*.

[54] Allen S. J., Watson J. J., Shoemark D. K., Barua N. U., Patel N. K. (2013). GDNF, NGF and BDNF as therapeutic options for neurodegeneration. *Pharmacology & Therapeutics*.

[55] Maranesi M., Parillo F., Leonardi L. (2016). Expression of nerve growth factor and its receptors in the uterus of rabbits: functional involvement in prostaglandin synthesis. *Domestic Animal Endocrinology*.

[56] Wessels J. M., Wu L., Leyland N. A., Wang H., Foster W. G. (2014). The brain-uterus connection: brain derived neurotrophic factor (BDNF) and its receptor (Ntrk2) are conserved in the mammalian uterus. *PLoS One*.

[57] Dolle L., Adriaenssens E., Yazidi-Belkoura I., Bourhis X., Nurcombe V., Hondermarck H. (2004). Nerve growth factor receptors and signaling in breast cancer. *Current Cancer Drug Targets*.

[58] Streiter S., Fisch B., Sabbah B., Ao A., Abir R. (2016). The importance of neuronal growth factors in the ovary. *Molecular Human Reproduction*.

[59] Pizot C., Boniol M., Mullie P. (2016). Physical activity, hormone replacement therapy and breast cancer risk: a meta-analysis of prospective studies. *European Journal of Cancer*.

[60] Sjogren L. L., Morch L. S., Lokkegaard E. (2016). Hormone replacement therapy and the risk of endometrial cancer: a systematic review. *Maturitas*.

[61] Garcia A. N., Depena C., Bezner K., Yin W., Gore A. C. (2018). The timing and duration of estradiol treatment in a rat model of the perimenopause: influences on social behavior and the neuromolecular phenotype. *Hormones and Behavior*.

[62] Kritzer M. F., Adler A., Bethea C. L. (2003). Ovarian hormone influences on the density of immunoreactivity for tyrosine hydroxylase and serotonin in the primate corpus striatum. *Neuroscience*.

[63] Garcia A. N., Bezner K., Depena C., Yin W., Gore A. C. (2017). The effects of long-term estradiol treatment on social behavior and gene expression in adult female rats. *Hormones and Behavior*.

[64] Bethea C. L., Kohama S. G., Reddy A. P., Urbanski H. F. (2016). Ovarian steroids regulate gene expression in the dorsal raphe of old female macaques. *Neurobiology of Aging*.

[65] Brambilla P., Perez J., Barale F., Schettini G., Soares J. C. (2003). GABAergic dysfunction in mood disorders. *Molecular Psychiatry*.

[66] Braden B. B., Kingston M. L., Koenig E. N., Lavery C. N., Tsang C. W. S., Bimonte-Nelson H. A. (2015). The GABA_A_ antagonist bicuculline attenuates progesterone-induced memory impairments in middle-aged ovariectomized rats. *Frontiers in Aging Neuroscience*.

[67] Gerhard D. M., Wohleb E. S., Duman R. S. (2016). Emerging treatment mechanisms for depression: focus on glutamate and synaptic plasticity. *Drug Discovery Today*.

[68] Jun C., Choi Y., Lim S. M. (2014). Disturbance of the glutamatergic system in mood disorders. *Experimental Neurobiology*.

[69] Marmiroli P., Cavaletti G. (2012). The glutamatergic neurotransmission in the central nervous system. *Current Medicinal Chemistry*.

[70] Jenkins T. A., Nguyen J. C., Polglaze K. E., Bertrand P. P. (2016). Influence of tryptophan and serotonin on mood and cognition with a possible role of the gut-brain axis. *Nutrients*.

[71] Amin Z., Canli T., Epperson C. N. (2005). Effect of estrogen-serotonin interactions on mood and cognition. *Behavioral and Cognitive Neuroscience Reviews*.

[72] Grimm J., Mueller A., Hefti F., Rosenthal A. (2004). Molecular basis for catecholaminergic neuron diversity. *Proceedings of the National Academy of Sciences of the United States of America*.

[73] Wang Z., Zhang A., Zhao B. (2016). GABA+ levels in postmenopausal women with mild-to-moderate depression: a preliminary study. *Medicine*.

[74] Li X., Wang W., Chen J. (2017). Recent progress in mass spectrometry proteomics for biomedical research. *Science China. Life Sciences*.

[75] Borahay M. A., Asoglu M. R., Mas A., Adam S., Kilic G. S., Al-Hendy A. (2017). Estrogen receptors and signaling in fibroids: role in pathobiology and therapeutic implications. *Reproductive Sciences*.

[76] Muhammad T., Ali T., Ikram M., Khan A., Alam S. I., Kim M. O. (2019). Melatonin rescue oxidative stress-mediated neuroinflammation/ neurodegeneration and memory impairment in scopolamine-induced amnesia mice model. *Journal of Neuroimmune Pharmacology*.

[77] Pariyar R., Yoon C. S., Svay T. (2017). *Vitis labruscana* leaf extract ameliorates scopolamine-induced impairments with activation of Akt, ERK and CREB in mice. *Phytomedicine*.

[78] Frick K. M., Zhao Z., Fan L. (2014). The epigenetics of estrogen. *Epigenetics*.

[79] Liang J., Shang Y. (2013). Estrogen and cancer. *Annual Review of Physiology*.

[80] Hervouet E., Cartron P. F., Jouvenot M., Delage-Mourroux R. (2013). Epigenetic regulation of estrogen signaling in breast cancer. *Epigenetics*.

[81] Mourad R., Hsu P. Y., Juan L. (2014). Estrogen induces global reorganization of chromatin structure in human breast cancer cells. *PLoS One*.

[82] Boyne D. J., Friedenreich C. M., McIntyre J. B., Stanczyk F. Z., Courneya K. S., King W. D. (2017). Endogenous sex hormone exposure and repetitive element DNA methylation in healthy postmenopausal women. *Cancer Causes & Control*.

[83] Ambatipudi S., Horvath S., Perrier F. (2017). DNA methylome analysis identifies accelerated epigenetic ageing associated with postmenopausal breast cancer susceptibility. *European Journal of Cancer*.

[84] Sanchez-Rodriguez M. A., Zacarias-Flores M., Arronte-Rosales A., Correa-Munoz E., Mendoza-Nunez V. M. (2012). Menopause as risk factor for oxidative stress. *Menopause: The Journal of The North American Menopause Society*.

[85] Kolesnikova L., Semenova N., Madaeva I. (2015). Antioxidant status in peri- and postmenopausal women. *Maturitas*.

[86] Kaur A., Jindal S., Kaur I. P., Chopra K. (2013). Effect of sesamol on the pathophysiological changes induced by surgical menopause in rodents. *Climacteric*.

[87] Cervellati C., Bergamini C. M. (2016). Oxidative damage and the pathogenesis of menopause related disturbances and diseases. *Clinical Chemistry and Laboratory Medicine*.

[88] Kouhestani S., Jafari A., Babaei P. (2018). Kaempferol attenuates cognitive deficit via regulating oxidative stress and neuroinflammation in an ovariectomized rat model of sporadic dementia. *Neural Regeneration Research*.

