# Supplementary material for: Total Polysaccharides of Lily Bulb Ameliorate Menopause-Like Behavior in Ovariectomized Mice: Multiple Mechanisms Distinct from Estrogen Therapy
Source: Oxid Med Cell Longev. 2019 Jul 25;2019:6869350. doi: 10.1155/2019/6869350 (PMC6683782; doi:10.1155/2019/6869350)
Supplement: Supplementary Materials — S-Table 1: list of various antibodies used in western blot analysis. S-Table 2: the involved gene names and the corresponding protein names in the KEGG pathways and volcano plots. S-Fig. 1: the effects of three doses of TPLB on body weight, uterine weight, and serum estradiol. (A) Variation of net body weight on the 14th day after OVX; (B) ratio of the uterus to body weight at the end of the 5-week treatment period; (C) serum estradiol levels. Data are expressed as mean ± SEM (n = 10) and examined with one-way ANOVA, followed by post hoc Dunnett's test: ∗ P < 0.05, ∗∗ P < 0.01, and ∗∗∗ P < 0.001 vs. the sham group; # P < 0.05, ## P < 0.01, and ### P < 0.001 vs. the OVX group. S-Fig. 2: the representational HPLC-DAD chromatograms for the measurement of glutamate and GABA (A) in the hypothalamus (B), hippocampus (C), and prefrontal cortex (D). S-Fig. 3: the effects of TPLB on the GABAergic system in the brain regions. GABA levels (n = 6) in the hypothalamus (A), hippocampus (B), and prefrontal cortex (C); representative images indicating the expression levels (n = 3) of GABAAα1 and GAD67 and their quantification analysis in the hypothalamus (D-E), hippocampus (F-G), and prefrontal cortex (H-I). Data are expressed as mean ± SEM and examined with one-way ANOVA, followed by post hoc Dunnett's test: ∗ P < 0.05, ∗∗ P < 0.01, and ∗∗∗ P < 0.001 vs. the sham group; # P < 0.05, ## P < 0.01, and ### P < 0.001 vs. the OVX group. S-Fig. 4: the effects of TPLB on the glutamatergic system in the brain regions. Glutamate levels (n = 6) in the hippocampus (A) and prefrontal cortex (B); representative images indicating the expression levels (n = 3) of NMDAR1 and CaMKII and their quantification analysis in the hippocampus (C-D) and prefrontal cortex (E-F). Data are expressed as mean ± SEM and examined with one-way ANOVA, followed by post hoc Dunnett's test: ∗ P < 0.05, ∗∗ P < 0.01, and ∗∗∗ P < 0.001; # P < 0.05, ## P < 0.01, and ### P < 0.001 vs. the OVX group. S-Fig. 5: the effects of [file 6869350.f1.pdf]

Supplementary materials

S-Table 1. List of various antibodies used in Western blot analysis

Primary Antibody	Species/clonality	Dilution	Manufacturer
Estrogen receptor α (ERα)	Rabbit monoclonal	1:1000	Invitrogen
Estrogen receptor β (ERβ)	Mouse monoclonal	1:4000	Abcam
Brain-derived neurotrophic factor (BDNF)	Rabbit polyclonal	1:1000	Santa Cruz
Nerve growth factor (NGF)	Rabbit polyclonal	1:1000	Abcam
Glial cell-derived neurotrophic factor (GDNF)	Rabbit polyclonal	1:1000	Abcam
Tyrosine hydroxylase (TH)	Rabbit monoclonal	1:1000	Santa Cruz
Serotonin transporter (ST)	Mouse monoclonal	1:1000	Santa Cruz
Gamma-aminobutyric acid A receptor alpha 1 ($GABA_{A\alpha1}$)	Goat monoclonal	1:1000	Santa Cruz
Glutamate decarboxylase (GAD 65)	Mouse monoclonal	1:2000	Abcam
N-methyl-D-aspartate receptor subunit 1 (NMDAR1)	Goat monoclonal	1:2000	Santa Cruz
Protein Kinase B (Akt)	Rabbit polyclonal	1:1000	CST
Phospho-Akt (p-Akt)	Rabbit polyclonal	1:1000	CST
cAMP-response element binding protein (CREB)	Rabbit monoclonal	1:1000	CST
Phospho-CREB (p-CREB)	Rabbit monoclonal	1:1000	CST
Extracellular signal–regulated kinases (ERK)	Rabbit polyclonal	1:1000	CST
Phospho- ERK (p- ERK)	Rabbit polyclonal	1:1000	CST
Ras	Rabbit polyclonal	1:1000	CST
Calmodulin-dependent protein kinase II (CaMKII)	Mouse monoclonal	1:2000	Santa Cruz
Phospho-CaMKII (p-CaMKII)	Mouse monoclonal	1:2000	Santa Cruz
Serotonin receptor 1A (SR-1A)	Rabbit monoclonal	1:1000	Santa Cruz
Glyceraldehyde-3-phosphate dehydrogenase (GAPDH)	Mouse monoclonal	1:5000	Immunoway

S-Table 2. The involved gene names and the corresponding protein names in the KEGG pathways and volcano plots

Protein name	Gene name
Fibroblast growth factor 12	Fgf12
Protein phosphatase 1B	Ppm1b
RAC-gamma serine/threonine-protein kinase	Akt3
GTPase NRas	Nras
Ankyrin-1	Ank1
Integrin alpha-V	Itgav
NADH dehydrogenase [ubiquinone] 1 alpha subcomplex subunit 7	Ndufa7
Cytochrome b-c1 complex subunit 9	Uqcr10
ATP synthase protein 8	ATP8
Metabotropic glutamate receptor 1	Grm1
Inositol 1,4,5-trisphosphate receptor type 1	Itpr1
Phosphoinositide phospholipase C	PLC
Dual specificity mitogen-activated protein kinase kinase 2	Map2k2
Ras-related protein Rab-5B	Rab5b
Transcription factor A, mitochondrial	Tfam
Serine protease HTRA2, mitochondrial	Htra2
Thymosin beta-4; Hematopoietic system regulatory peptide	Tmsb4x
Core histone macro-H2A.2; Core histone macro-H2A	H2afy2
Histone H3; Histone H3.2; Histone H3.1	Hist1h3e;Hist2h3b;Hist1h3b;H3f3a;Hist1h3i;Hist1h3a
Histone H2B; Histone H2B type 1-P;Histone H2B type 1-K;Histone H2B type 1-C/E/G; Histone H2B type 2-B;Histone H2B type 1-H;Histone H2B type 1-B;Histone H2B type 1-M;Histone H2B type 1-F/J/L	Hist1h2bj;Hist1h2bk;Hist1h2bm;Hist1h2br;LOC665622;Hist1h2bp;Hist1h2bc;Hist2h2bb;Hist1h2bh;Hist1h2bb;Hist1h2bf
Histone H4	Hist2h4;Hist1h4a
Histone H2B type 3-A; Histone H2B type 3-B;Histone H2B type 2-E	Hist3h2ba;Hist3h2bb;Hist2h2be
Alpha-actinin-2	Actn2
Alpha-actinin-4	Actn4
Calcium/calmodulin-dependent protein kinase kinase 1	Camkk1
Guanine nucleotide-binding protein G(I)/G(S)/G(O) subunit gamma-4; Guanine nucleotide-binding protein subunit gamma	Gng4

Chromodomain-helicase-DNA-binding protein 4	Chd4
Calcium/calmodulin-dependent protein kinase type IV	Camk4
Metabotropic glutamate receptor 1	Grm1
Inositol 1,4,5-trisphosphate receptor type 1	Itpr1
Phosphoinositide phospholipase C	Plcb4
Peptidyl-prolyl cis-trans isomerase FKBP4; Peptidyl-prolyl cis-trans isomerase FKBP4, N-terminally processed; Peptidyl-prolyl cis-trans isomerase	Fkbp4
RAC-alpha serine/threonine-protein kinase	Akt1
Gap junction alpha-1 protein; Gap junction protein	Gja1
Tubulin alpha-8 chain	Tuba8
Sodium/potassium-transporting ATPase subunit beta-2	Atp1b2
Glutamate receptor 2	Gria2
Amino acid transporter; Excitatory amino acid transporter 1	Slc1a3
Homer protein homolog 2	Homer2

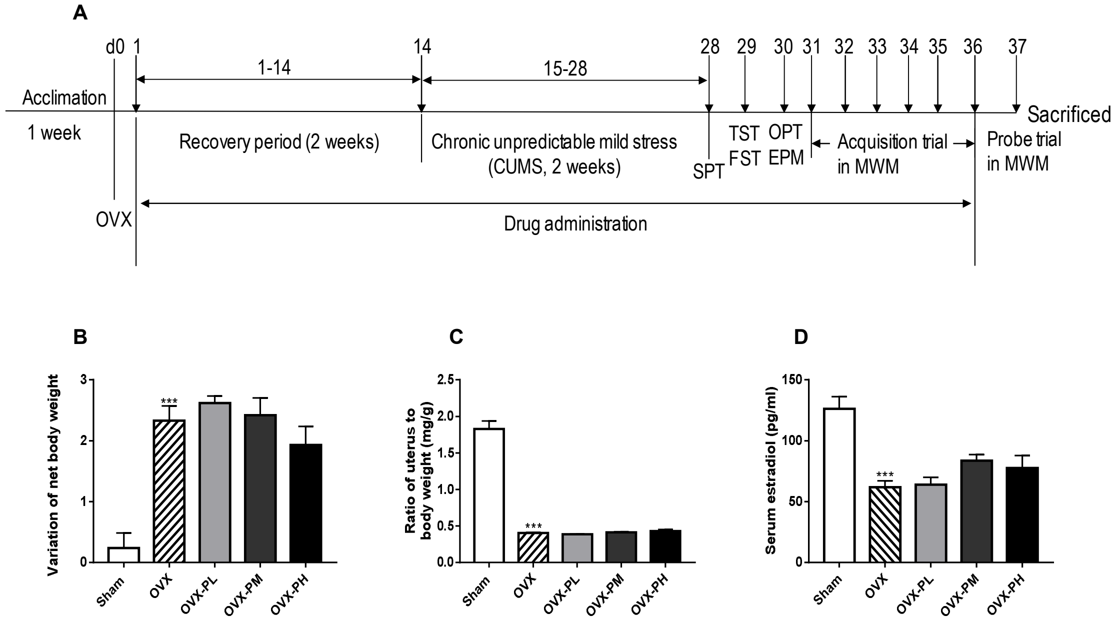

S-Fig. 1. The effects of three doses of TPLB on body weight, uterine weight and serum estradiol. (A) Variation of net body weight on the 14th day after OVX; (B) Ratio of uterus to body weight at the end of the 5-week treatment period; (C) Serum estradiol levels. Data are expressed as mean ± SEM (n = 10) and examined with one-way ANOVA, followed by post hoc Dunnett's test: * *P* <0.05, ** *P* <0.01, *** *P* <0.001 vs the Sham group; # *P* <0.05, ## *P* <0.01, ### *P* <0.001 vs the OVX group.

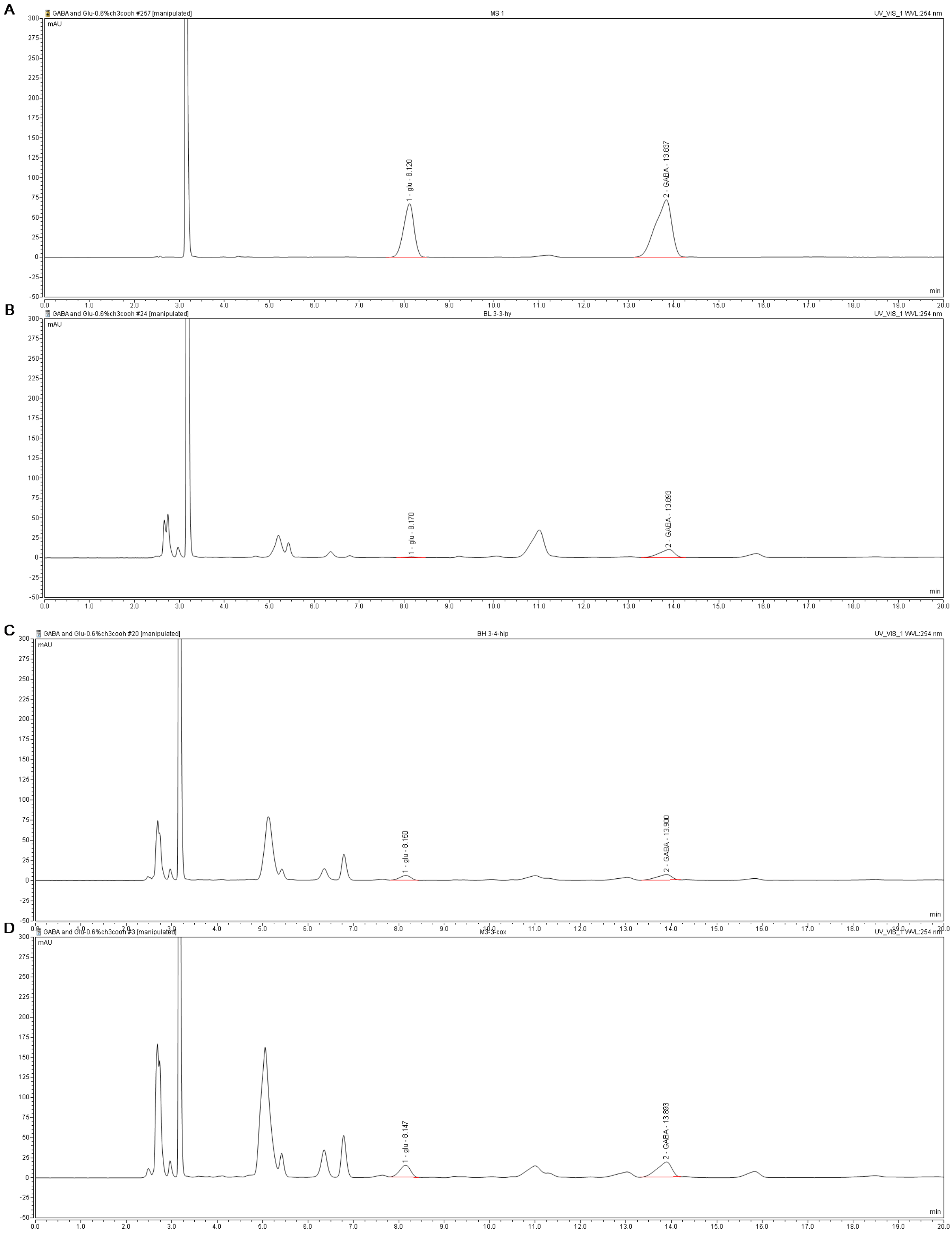

S-Fig. 2. The representational HPLC-DAD chromatograms for measurement of Glutamate and GABA (A) in hypothalamus (B), hippocampus (C) and prefrontal cortex (D).

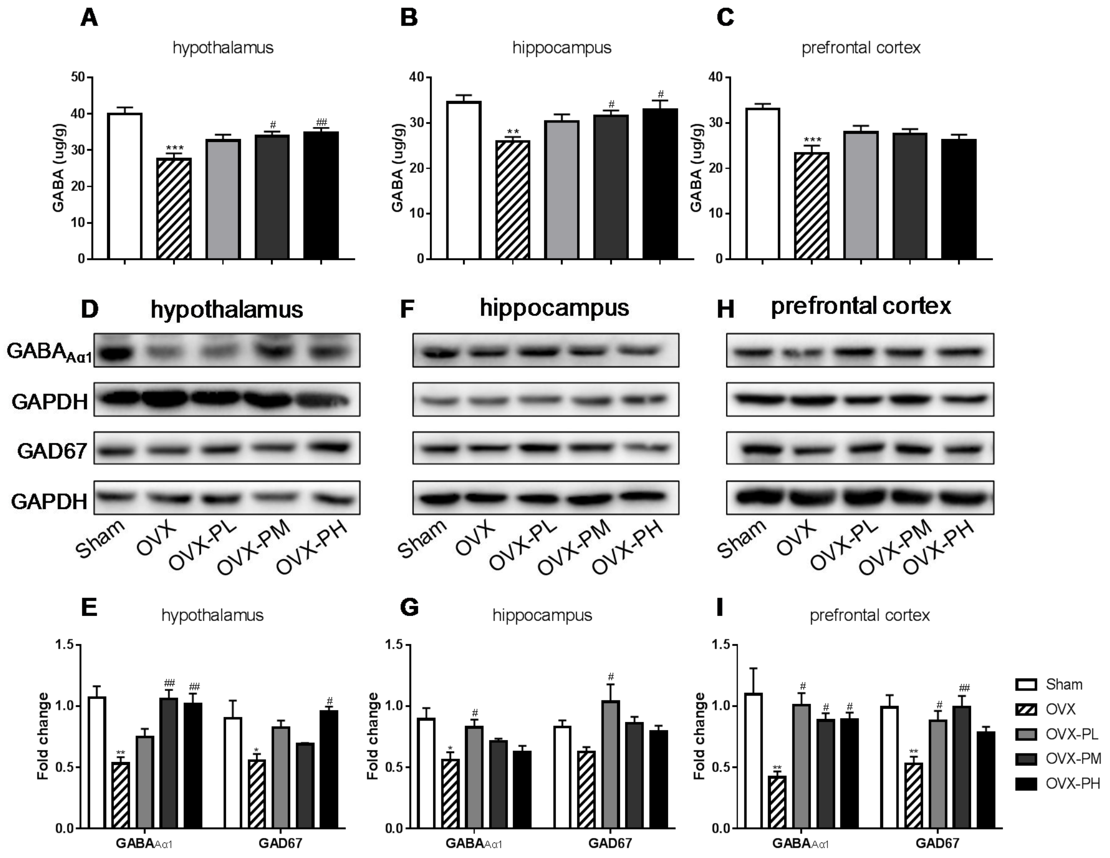

S-Fig. 3. The effects of TPLB on the GABAergic system in brain regions. GABA levels (n = 6) in hypothalamus (A), hippocampus (B) and prefrontal cortex (C); representative images indicating the expression levels (n = 3) of $GABA_{A\alpha1}$ and GAD67 and their quantification analysis in hypothalamus (D-E), hippocampus (F-G) and prefrontal cortex (H-I). Data are expressed as mean ± SEM and examined with one-way ANOVA, followed by post hoc Dunnett's test: * *P* <0.05, ** *P* <0.01, *** *P* <0.001 vs the Sham group; # *P* <0.05, ## *P* <0.01, ### *P* <0.001 vs the OVX group.

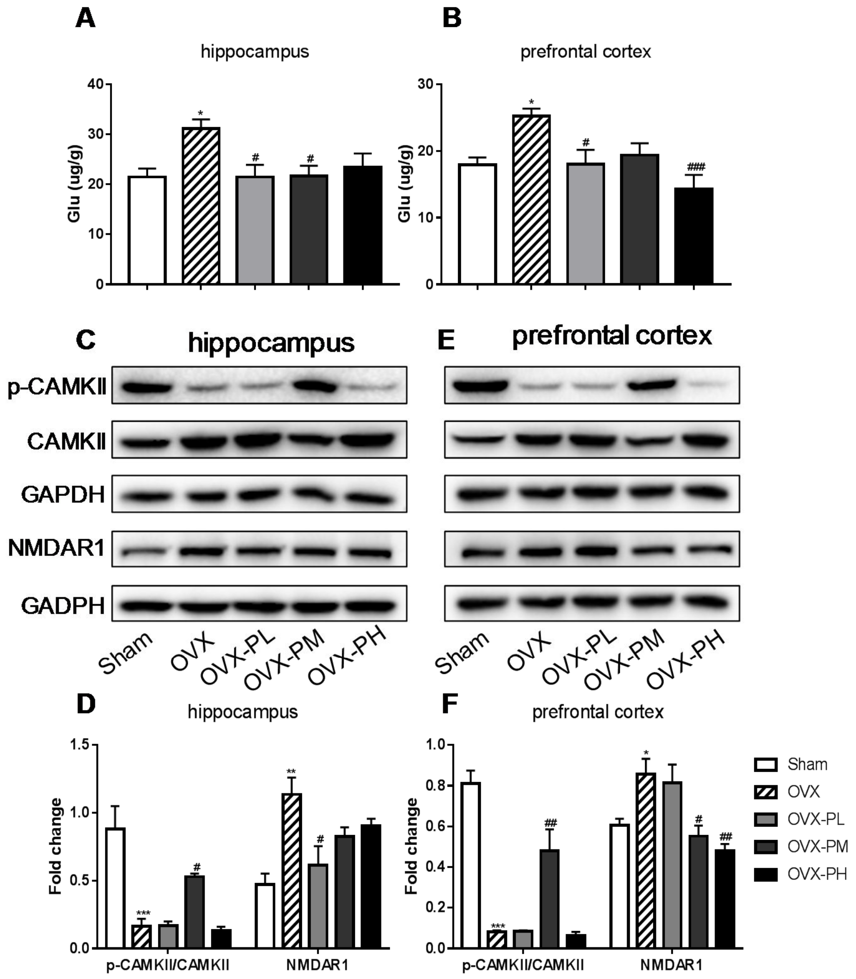

S-Fig. 4. The effects of TPLB on the on the glutamatergic system in brain regions. Glutamate levels (n = 6) in hippocampus (A) and prefrontal cortex (B); representative images indicating the expression levels (n = 3) of NMDAR1 and CaMKII and their quantification analysis in hippocampus (C-D) and prefrontal cortex (E-F). Data are expressed as mean ± SEM and examined with one-way ANOVA, followed by post hoc Dunnett's test: * P <0.05, ** P <0.01, *** P <0.001; # P <0.05, ## P <0.01, ### P <0.001 vs the OVX group.

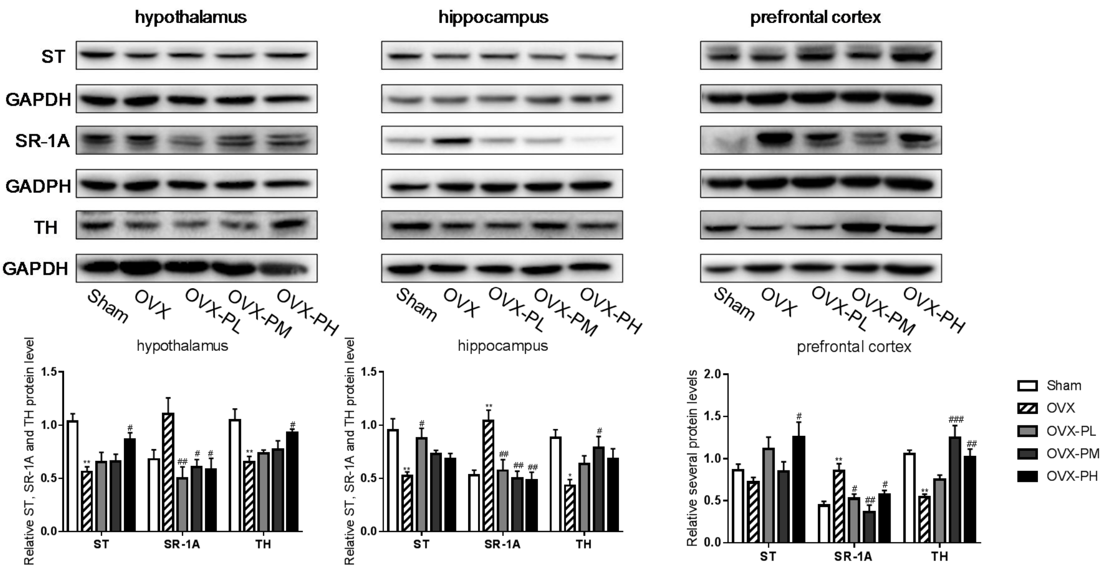

S-Fig. 5 The effects of three doses of TPLB on the serotonergic and catecholaminergic systems in brain regions. Representative images indicating the expression levels of ST and SR-1A, TH and their quantification analysis in hypothalamus (A-B), hippocampus (C-D) and prefrontal cortex (E-F). Data are expressed as mean ± SEM (n = 3) and examined with one-way ANOVA, followed by post hoc Dunnett's test: * P <0.05, ** P <0.01, *** P <0.001; # P <0.05, ## P <0.01, ### P <0.001 vs the OVX group.

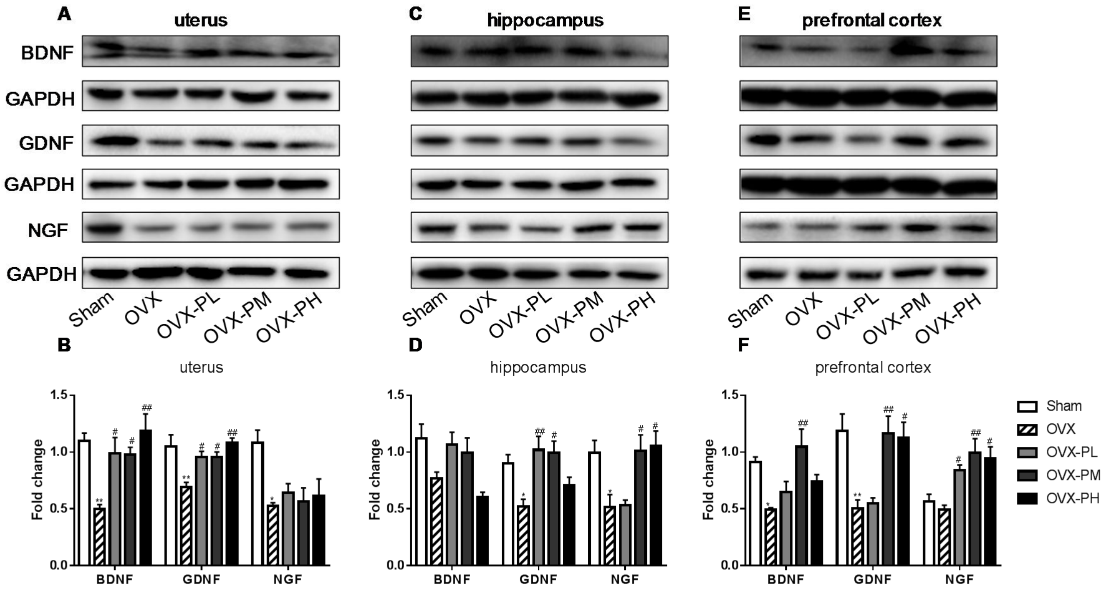

S-Fig. 6 The effects of three doses of TPLB on the expression levels of neurotrophins, BDNF, GDNF and NGF, in uterus, hippocampus and prefrontal cortex. Representative images indicating the expression levels of three neurotrophins and their quantification analysis in uterus (A-B), hippocampus (C-D) and prefrontal cortex (E-F). Data are expressed as mean ± SEM (n = 3) and examined with one-way ANOVA, followed by post hoc Dunnett's test: * P <0.05, ** P <0.01, *** P <0.001; # P <0.05, ## P <0.01, ### P <0.001 vs the OVX group.

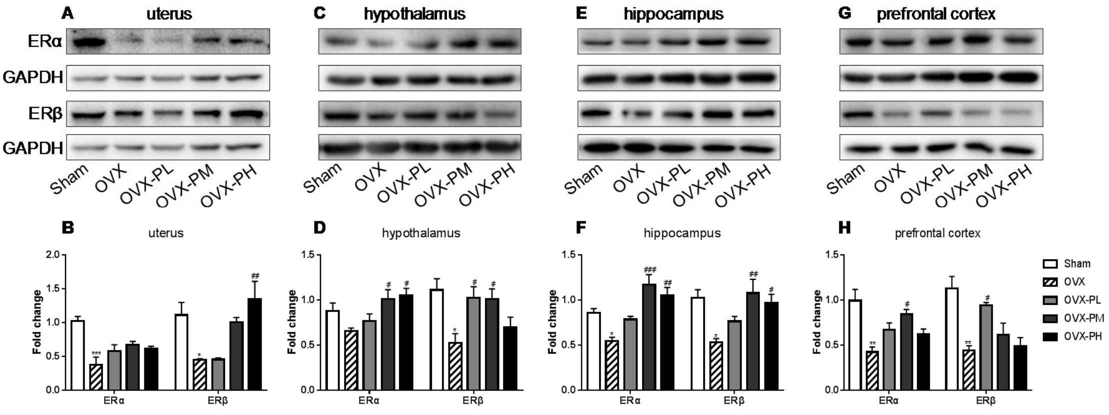

S-Fig. 7. The effects of three doses of TPLB on the expression levels of estrogen receptors, ERα and ERβ, in uterus and brain regions. Representative images indicating the expression levels of ERα and ERβ and their quantification analysis in uterus (A-B), hypothalamus (C-D), hippocampus (E-F) and prefrontal cortex (G-H). Data are expressed as mean ± SEM (n = 3) and examined with one-way ANOVA, followed by post hoc Dunnett's test: * P <0.05, ** P <0.01, *** P <0.001; # P <0.05, ## P <0.01, ### P <0.001 vs the OVX group.

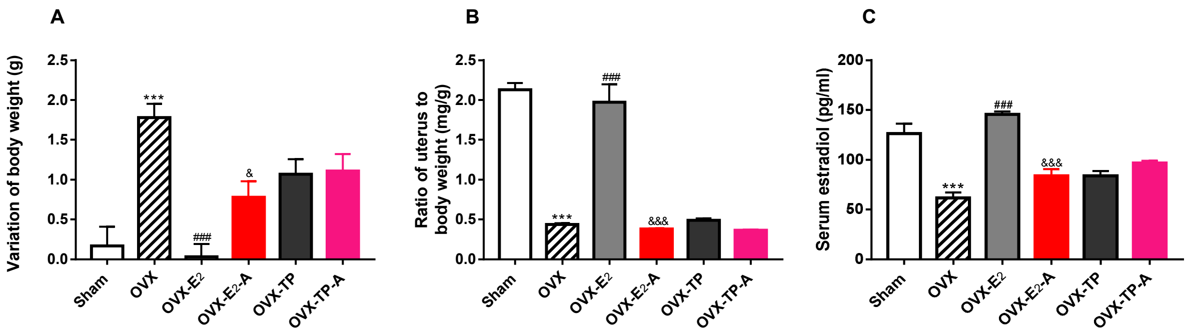

S-Fig. 8. The effects of estradiol and TPLB on body weight, uterine weight and serum estradiol after co-administration with ICI182,780. (A) Variation of net body weight on the 14th day after OVX; (B) Ratio of uterus to body weight at the end of the 5-week treatment period; (C) Serum estradiol levels. Data are expressed as mean ± SEM (n = 10) and examined with one-way ANOVA, followed by post hoc Sidak's multiple comparisons test: * *P* <0.05, ** *P* <0.01, *** *P* <0.001 vs the Sham group; # *P* <0.05, ## *P* <0.01, ### *P* <0.001 vs the OVX group; & *P* <0.05, && *P* <0.01, &&& *P* <0.001 vs the E_2 group or TP group.

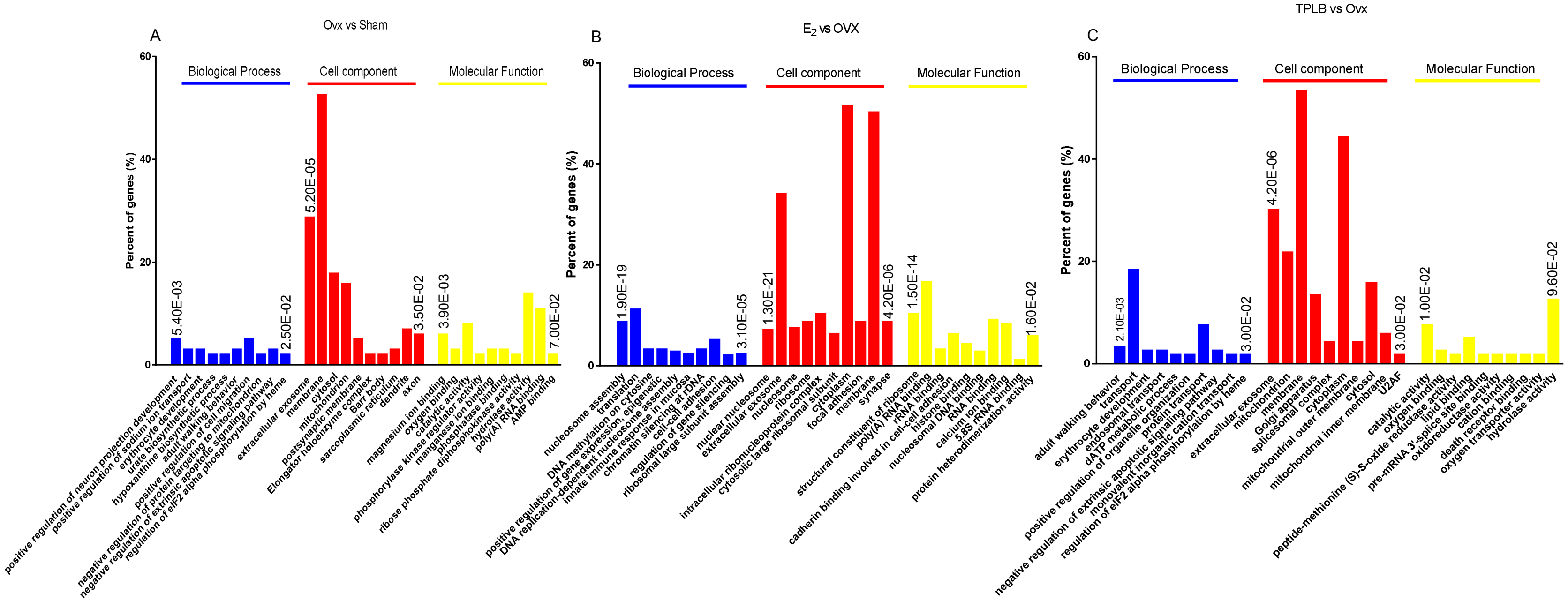

S-Fig. 9. The ten most significantly enriched three terms in gene ontology (GO): biological process, cell component, and molecular function.
